# Comprehensive metabolome characterization of leaves, internodes, and aerial roots of *Vanilla planifolia* by untargeted LC–MS and GC × GC–MS

**DOI:** 10.1002/pca.3414

**Published:** 2024-07-21

**Authors:** Falco Beer, Christoph H. Weinert, Johannes Wellmann, Silke Hillebrand, Jakob Peter Ley, Sebastian T. Soukup, Sabine E. Kulling

**Affiliations:** ^1^ Department of Safety and Quality of Fruit and Vegetables, Max Rubner‐Institut Federal Research Institute of Nutrition and Food Karlsruhe Germany; ^2^ Symrise AG Holzminden Germany

**Keywords:** metabolite profiling, Orchidaceae, phenolics, *Vanilla planifolia*, vanillin

## Abstract

**Introduction:**

Untargeted metabolomics is a powerful tool that provides strategies for gaining a systematic understanding of quantitative changes in the levels of metabolites, especially when combining different metabolomic platforms. Vanilla is one of the world's most popular flavors originating from cured pods of the orchid *Vanilla planifolia* . However, only a few studies have investigated the metabolome of *V. planifolia* , and no LC–MS or GC–MS metabolomics studies with respect to leaves have been performed.

**Objective:**

The aim of the study was to comprehensively characterize the metabolome of different organs (leaves, internodes, and aerial roots) of *V. planifolia* .

**Material and Methods:**

Characterization of the metabolome was achieved using two complementary platforms (GC × GC–MS, LC‐QToF‐MS), and metabolite identification was based on a comparison with in‐house databases or curated external spectral libraries.

**Results:**

In total, 127 metabolites could be identified with high certainty (confidence level 1 or 2) including sugars, amino acids, fatty acids, organic acids, and amines/amides but also secondary metabolites such as vanillin‐related metabolites, flavonoids, and terpenoids. Ninty‐eight metabolites showed significantly different intensities between the plant organs. Most strikingly, aglycons of flavonoids and vanillin‐related metabolites were elevated in aerial roots, whereas its *O*‐glycoside forms tended to be higher in leaves and/or internodes. This suggests that the more bioactive aglycones may accumulate where preferably needed, e.g. for defense against pathogens.

**Conclusion:**

The results derived from the study substantially expand the knowledge regarding the vanilla metabolome forming a valuable basis for more targeted investigations in future studies, e.g. towards an optimization of vanilla plant cultivation.

## Abstract 2

The aim of the study was to comprehensively characterize the metabolome of leaves, internodes and aerial roots of V. planifolia using two complementary platforms (GC × GC–MS, LC–QToF–MS). 127 metabolites were identified with certainty, and 98 thereof showed significant differences between the plant organs. Whereas the O‐glycosides of flavonoids and vanillin‐related metabolites tended to be higher in leaves and/or internodes, their aglycons were elevated in aerial roots suggesting that the more bioactive aglycones may accumulate where preferably needed, e.g. for pathogen defense.

## 1 INTRODUCTION

Vanilla is one of the world's most popular flavors extracted from cured beans of the orchid genus *Vanilla*, which belongs to the family of Orchidaceae.^1^ Only two species, *Vanilla (V.) planifolia* and *Vanilla x tahitensis*, which are among the most expensive condiments on the market, are cultivated on a large scale for commercial purposes.^2^ Natural vanilla is well‐known for its characteristic flavor due to a complex mixture of more than 250 compounds. Vanillin can be regarded as a key aroma compound in vanilla, and with respect to vanilla pods, it accounts for 1–8% of dry matter^3^ and for 80% of total aromatics.^4^ It has been widely used not only for flavoring and as a fragrance but also as a food‐preserving agent due to its antimicrobial and antioxidative properties.^5^, ^6^

Metabolomics has become a powerful tool that can be used for in‐depth mining of plant metabolic diversity and function.^7^ The coverage of the metabolome can be significantly improved by combining multiple analytical techniques, such as liquid chromatography–mass spectrometry (LC–MS) and gas chromatography–mass spectrometry (GC–MS).^8^, ^9^

With respect to vanilla plants and vanilla pods, only a few metabolomics studies are available in the literature. For example, Busconi et al^10^ characterized the pods of *V. x tahitensis* by LC–MS‐based metabolite profiling approach for traceability purposes. In this study, 260 compounds were annotated including previously unreported phenolic compounds such as flavonoids, stilbenes, and lignans. Regarding the leaf metabolome only limited information is available. The biological variation of the leaf metabolome of *V. planifolia* was studied using nuclear magnetic resonance (NMR) spectroscopy with regard to the development stage, season, and time of day,^11^ and with respect to intra‐species differences under virus infection conditions.^12^ But, in both studies only a small number of selected metabolites were identified. In more recent work, Leyva et al^13^ characterized the leaf and stem metabolome of *V. planifolia* by means of NMR‐metabolomics, and 36 distinct metabolites were reported. However, so far, no respective LC–MS‐ or GC–MS‐metabolomics‐based studies have been conducted for vanilla leaves.

The aim of our study was to explore the metabolome of different organs (leaves, internodes, and aerial roots) of *V. planifolia* using two complementary metabolomic platforms (GC × GC–MS, LC‐QToF‐MS). At this, we expected to gain new insights into the organ‐specific metabolome of *V. planifolia* with a special focus on vanillin biosynthesis,^3^, ^14^, ^15^ which has not yet been fully elucidated and is still a matter of debate.

## 2 MATERIAL AND METHODS

### 2.1 Chemicals

Solvents, eluents, and additives used for LC–MS analysis were of MS grade and purchased from Merck/Sigma‐Aldrich (Darmstadt, Germany). *N*‐Methyl‐*N*‐(trimethylsilyl)trifluoroacetamide (MSTFA) was purchased from Carl Roth (Karlsruhe, Germany); methoxylamine hydrochloride and pyridine were from Merck/Sigma‐Aldrich (Darmstadt, Germany). Reference compounds were purchased from different commercial suppliers, e.g. Sigma‐Aldrich (Taufkirchen, Germany), Phytolab (Vestenbergsgreuth, Germany), and Biosynth (Compton, Berkshire, UK). More detailed information is provided in the Supporting Information Tables S1 and S2.

### 2.2 Plant cultivation and sampling

Authentic plant material was purchased from Orchideen Roellke. Vanilla species originated from virus‐free meristem culture in 1985. The cultivation of the one‐year‐old vanilla plants took place in a cultivation room at Symrise AG (Holzminden, Germany). The temperature was set to 26°C and the average humidity was adjusted to 70%. The LED lighting was from Valoya LED (BX‐Series NS1‐ Valoya OY, Helsinki, Finland) comprising a balanced sun spectrum for research, and the day/night cycle was set to 12 h each. The luminosity has been reduced to 300 μmol m^−2^ s^−1^. The orchid substrate compo sana (Compo GmbH, Münster, Germany) was used, and fertilization was carried out once a week with the orchid fertilizer of Wuxal (Hauert Manna GmbH, Nürnberg, Germany) in 10‐fold dilution.

The plant material was selected and harvested with a pair of secateurs (previously disinfected) and transported from the cultivation room to the laboratory in a paper bag. The length of the harvested plant was determined and then divided into three equal parts. Mixed samples were taken from the lower, middle, and upper third of the plant, which were then divided into leaves, internodes, and aerial roots.

The relatively large succulent leaves were separated from the stems without the nodules. The samples were wrapped in aluminum foil and frozen in liquid nitrogen (ca. three to four leaves). Likewise, the aerial roots (ca. three to four) were separated from the stems and frozen as described above. The internodes (ca. eight pieces) were separated, cut into approximately 2 cm long pieces, and rapidly frozen in liquid nitrogen as described above. The frozen samples were stored at −80°C until lyophilization.

### 2.3 Plant sample processing

#### 2.3.1 Freeze‐drying and pre‐shredding

The freeze‐drying process was completed after 72 h at a cooling trap temperature of ca. ‐80°C and a vacuum of < 1 mbar. The freeze‐drying cabinet was covered with aluminum foil to minimize light exposure. After freeze drying samples were ground to powder for 3 min at 20,000 rpm without further cooling. For this purpose, the IKA tube mill control (Staufen, Germany) was used. The powdered samples were transferred to falcon tubes, filled with argon, closed tightly, and stored at −20°C.

#### 2.3.2 Fine grinding

The frozen and pre‐shredded plant material was transferred on dry ice from the cultivation side (Holzminden) to Max Rubner‐Institut (Karlsruhe) and was stored at the destination at −80°C. Samples were finely ground using a ball mill from Retsch (Haan, Germany) until they reached a fine powder. Grinded samples were stored at −80°C until further processing. From each grinded sample three separate 20 mg (± 0.2 mg) aliquots were exactly weighed into 2 ml tubes. Due to limited sample material, in the case of aerial roots, aliquots of 10 mg (± 0.1 mg) were taken. Aliquots were stored at −80°C until further analysis.

#### 2.3.3 Quality control (QC) sample preparation

For quality assurance purposes a quality control (QC) sample was prepared. Comparable amounts of leaf, internode, and, to a slightly lesser extent, aerial root material were mixed in a 5 ml tube. Following thorough homogenization of the mixed QC sample using an overhead shaker and a vortex mixer, 20 mg (± 0.2 mg) aliquots were taken. Aliquots were stored at −80°C until analysis.

### 2.4 Untargeted UHPLC–QToF–MS analysis

#### 2.4.1 Internal standard (IS) mix preparation

Eighteen reference compounds were used as internal standards (IS) and for monitoring the chromatographic and mass spectrometric performance during measurement. Detailed information with regard to the authentic standards and the preparation of standard solutions are given in Supporting Information Table S1 and Data S3, respectively.

#### 2.4.2 Sample preparation for UHPLC–QToF–MS measurements

Based on the results of a preliminary experiment using vanilla leaf samples a sample/solvent ratio of 1:30 (ω[mg]/v[μl]) was chosen for this study. Frozen vanilla samples (leaf, internode, aerial root, or QC aliquots previously weighted in 2 ml tubes) were kept at room temp. For 15 minutes to allow for initial thawing. Subsequently, an appropriate volume of ice‐cooled pure methanol (600 μl for 20 mg aliquots or 300 μl for 10 mg aliquots) was added. Then, samples were spiked with respective aliquots of IS mix 1 and 2 (each 24 μl for 20 mg aliquots or each 12 μl for 10 mg aliquots) corresponding to an 1:25 (v/v) dilution of the IS working solutions (see Supporting Information Data S3). Moreover, five solvent blanks were prepared by pipetting 600 μl methanol into each 2 ml tube. The general procedure for controls was the same as for the study and QC samples, but instead of IS working solutions appropriate volumes of water and methanol were added. After thorough homogenization by a vortex mixer (5 s), controls, and matrix samples were incubated on a thermomixer (35°C, 1,400 rpm, 15 min). Following centrifugation (4°C, 16,100 *g*, 5 min) supernatants were purified using PTFE syringe filters (4 mm i.d., 0.2 μm) from Phenomenex (Aschaffenburg, Germany). Resulting filtrates were homogenized on a vortex mixer for 5 s. Final solutions were transferred to LC–MS certified TruView vials (Waters; Eschborn, Germany) and stored at 4°C until analysis. All samples were freshly prepared on each measurement day and measured within 24 h.

#### 2.4.3 UHPLC–QToF–MS measurements

Measurements were performed on an UHPLC–QToF–MS instrument: An Infinity 1290 LC system from Agilent Technologies (Waldbronn, Germany), equipped with a binary high gradient pump with degasser, a column oven, an autosampler, and a DAD, coupled to a high‐resolution TripleTOF 5600 tandem mass spectrometer (AB Sciex, Darmstadt, Germany) comprising a DuoSpray ion source.

Chromatographic separation was performed on a Waters Acquity UPLC HSS T3 Premier column (2.1 × 150 mm; 1.8 μm) with a guard column of the same material. The column oven temperature was set to 40°C. 2 mM ammonium formate with 0.05% (v/v) formic acid (A) and acetonitrile (B) were used as eluents at a flow rate of 0.4 ml/min, and the following gradient was applied: 0.0 min, 3% B; 3.0 min, 3% B; 14.0 min, 99% B; 20.0 min, 99% B; 20.5 min, 3% B; 25.5 min, 3% B. Injection volume was 2 μl. A mixture of H_2_O/acetonitrile (50:50, v/v) was used as injection needle rinsing solution. Analytes were detected by mass spectrometer as described below.

Eluate from the UHPLC was directed to the electrospray ionization probe of the DuoSpray ion source, and the atmospheric pressure chemical ionization probe was used for MS mass calibration by infusing a commercially available calibration solution after each eighth run. Ion source parameters were set as follows: temperature 550°C, curtain gas 45 psi, ion source gas‐1 and ‐2 both 60 psi. Full scan (m/z 80–1,500) and MSMS experiments (m/z 50–1,500) were conducted in information‐dependent acquisition mode. Accumulation time was set to 100 ms and 25 ms for full scan and product ion scan, respectively. Collision energies for product ion experiments were set to 35 ± 15 V and −35 ± 15 V, respectively. Mass spectra were recorded based on the top three candidate ions applying an intensity threshold of 450 cps and an m/z tolerance of 25 ppm. Nitrogen was used as collision gas.

The entire sample set was analyzed by LC‐QToF‐MS in negative and positive polarity. Due to limited sample amount availability, two individual samples (one leaf sample and one aerial root sample) could not be analyzed in positive mode. Information on the sample sequence that ensured robust data acquisition can be found in Supporting Information Data S3.

#### 2.4.4 UHPLC–QToF–MS data processing

The open‐access software MS‐DIAL (version 4.90) was used for data processing comprising the steps of peak detection, centroidization, identification, and alignment. Prior to data processing, equilibration QC samples and post‐calibration QC samples were excluded. Raw files were converted into ABF format using the Reacys AbfConverter (version 1.03.7328). Data collection parameters (centroidization) were set to 0.025 Da and 0.075 Da for MS1 and MS2 tolerance, respectively. For peak detection, the Savitzky Golay filter was applied with a smoothing level of 2 and a minimum peak width of 7 scans. The minimum peak height was set to 1,000 and the mass slice width was 0.075 Da. For alignment the blank filter option was enabled; all features with a sample average/blank average intensity of 3 or less were automatically eliminated from the feature list. The parameter ´N% detected in at least one group´ was set to 75%, i.e. any feature had to be present in at least 75% of all samples in at least one group of the entire sample group set not to be excluded from the feature list.

#### 2.4.5 Monitoring and quality assurance for UHPLC–QToF–MS measurements

Peak intensities and retention times of IS‐spiked samples were monitored in the regularly injected QC and potential drift effects were evaluated. Base peak chromatograms and LC pressure profiles were checked visually to verify the reproducibility of the measurement. For intensity drift verification the following procedure was conducted. An intensity drift correction was performed using the LOWESS algorithm in MS‐DIAL. The corresponding drift‐correction plots were evaluated, and the drift‐corrected and the uncorrected datasets were compared with each other regarding data quality. Therefore, the distribution of retention times, m/z values, and intensities were visualized and evaluated. In addition, a principal component analysis (PCA) was performed in JMP (version 17.0.0) using the adjusted feature lists of drift‐corrected and uncorrected dataset (see section 2.6.2). For evaluation, the variances within the QC group in the generated score plots were assessed.

### 2.5 Untargeted GC × GC–MS analysis

#### 2.5.1 Sample extraction, evaporation, and derivatization

On the day of analysis, the study samples as well as the QC sample aliquots were extracted twice with pure methanol at 35°C for 10 min using a sample/solvent ratio of 1:75 (ω[mg]/v[μl]). Aliquots of the supernatants as well as a mixture of seven unlabeled IS were then transferred to glass vials with micro‐inserts and evaporated in a rotary vacuum concentrator. The dried residues were derivatized according to the common methoximation/trimethylsilylation protocol. For details see Supporting Information Table S4.

#### 2.5.2 GC × GC–MS measurements

The untargeted GC × GC–MS analysis was done with a single‐oven GC‐qMS system (GC‐2010/QP2010 Ultra; Shimadzu, Kyoto, Japan) equipped with a cryogenic ZX‐2 modulator (ZOEX, Houston, USA) and a PAL3 RTC autosampler (CTC, Zwingen, Switzerland). The instrument was controlled by the software Chronos 5.0, GCMSsolution 4.45, and Evolution Workstation 4.6.7. The GC × GC method and all further instrumental settings are compiled in Supporting Information Table S5. Twenty‐nine study samples and ten QC samples were prepared and analyzed as one batch. The batch consisted of six equilibration runs with matrix samples followed by six blocks of four to five study samples, with QC sample injections in between as well as at the beginning and the end. Additionally, one RI‐QC sample (spiked with retention index marker mixtures, i.e. alkanes and fatty acid methyl esters) and a reagent blank were analyzed. One study sample was lost during derivatization.

#### 2.5.3 GC × GC–MS data processing

The acquired GC × GC–MS data were processed in a two‐step procedure. Firstly, peak integration and automated spectral matching against an in‐house library was performed using the Postrun Analysis module of GCMS Solution and an MS Excel macro which resulted in one text file per raw data file containing the peak data and the corresponding spectra. Afterward, these text files were further processed using R scripts developed in‐house. Essentially, this post‐processing comprised the import of the peak lists, data aggregation, noise filtering, alignment, peak merging, and QC sample‐based drift correction. This approach has been described in detail by Egert et al.^16^

### 2.6 Data analysis

#### 2.6.1 Metabolite identification

Reporting of the identification results was according to the four confidence levels system proposed by Sumner et al^17^ with minor modifications. One feature was classified as unequivocally identified (confidence level 1), if precursor ion mass, retention time, and MS2 spectrum matched to the data of a respective reference compound in the in‐house database. As no spiking experiments were performed in this study, metabolites were assigned to level 1* instead of level 1. Assignment of features/analytes by level 2 was used when spectral matching was performed with an external database. Level 3 was applied, when either no spectral matching was possible or the feature/analyte could be assigned to one specific compound class.

##### Metabolite identification based on LC–MS metabolomics

Automated feature annotation was performed using the ´post‐identification´ tool in MS‐DIAL by applying an in‐house LC–MS spectral library that comprised m/z values, ion species, and retention times of ca. 550 reference compounds originating from a broad spectrum of compounds, among them both, primary metabolites (e.g. sugar compounds, amino acids, amines, organic acids) and plant secondary metabolites (e.g. phenolic acids, flavonoids). Further details of the metabolite identification procedure are described in Supporting Information Data S6.

##### Metabolite identification based on GC × GC–MS metabolomics

During automated data processing (see section 2.5.3), metabolite identification was performed based on an in‐house library. The results of this automated annotation were checked manually. To identify further compounds of interest, a manual spectral matching was done against the NIST2017 library (without using the retention index information) and the Fiehn library (including the FAME‐based retention indices).

#### 2.6.2 Statistical analysis

##### Adjustment of data matrix prior to statistical analysis

With regard to GC × GC–MS data, a detection abundance filter corresponding to the alignment filter in MS‐DIAL (see section 2.4.4) was applied, and analytes present in the solvent blanks were eliminated after manual check of the GC × GC chromatograms. Then, all features/analytes (for LC and GC data sets) showing a coefficient of variance (CV) in the QC group of greater than 30% and/or a QC missing rate of greater than 25% were treated as non‐valid and thus eliminated from the data sets.^18^ In the respective feature lists (all reported features or identified metabolites) random intensities for missing values (not detected) were imputed by values in a defined numerical range depending on the analytical technique used. In the case of LC–MS data, the lower limit was set to the global intensity minimum of the feature matrix, and the upper limit was defined as the feature‐specific 1% percentile intensity. Based on long‐term experience for GC–MS data, the lower and the upper limit were set to 20,000 and 100,000, respectively.

##### Univariate and multivariate statistics

The dataset was analyzed in two ways. First, in the case of the identified metabolites (see section 2.6.1), peak intensities were compared between leaves, internodes, and aerial roots using univariate statistics. A Kruskal‐Wallis test was performed, followed by a Steel Dwass test as a post‐doc test. The results of a statistical test were assessed as significant if the calculated p‐value was smaller than the significance level of α = 0.05.

In addition, principal component analyses were conducted for the feature lists only containing valid features (see first paragraph). All statistical analyses were performed using the software JMP (version 17.0.0).

## 3 RESULTS AND DISCUSSION

### 3.1 Quality assurance of metabolomic analysis

During metabolomic analyses, a large number of matrix‐loaded samples are consecutively injected, which can affect analytical performance during the progression of measurement, especially due to contamination of the column and/or the MS source. This can lead to a drift of masses, intensities, and/or retention times. Potential effects on data quality need to be monitored and if necessary corrected.

For this purpose, a detailed validation of analytical performance (reproducibility and potential drift effects) was conducted. In the case of LC–MS analyses the procedure was as follows. An automatic external mass calibration was performed after each eighth run, to ensure good mass accuracies during measurements. In order to test for intensity and retention time drift, QC samples were regularly injected during each measurement series. All study and QC samples were spiked with IS belonging to different compound classes with retention times between 1 and 8 min, molecular masses from 100 to 400 Da, and corresponding expected intensities in the range of 10^2^ to 10^5^ (see Supporting Information Table S1). Retention times and intensities of IS were monitored in selected QC samples (one QC sample per block of eight) as well as in the IS mix solution in pure solvent. No indications for significant drift of either retention times or intensities were observed. The calculated CV for intensities and retention times were lower than 30% and 1%, respectively, with the exception of a few polar IS eluting in the injection peak at ca. 1.0 min ([^13^C_5_, ^15^N]valine, D‐[UL‐^13^C_6_]mannitol, D‐pinitol, and methyl‐α‐D‐glucopyranoside) with CV values of 30–40% and 1.0–1.6%, respectively. The maximum deviation of retention time was lower than 0.05 min for all IS with one exception (methyl‐deoxyribose; 0.1 min). The monitored pressure profiles and base peak chromatograms were comparable within each measurement series thus demonstrating good reproducibility.

**FIGURE 1:**
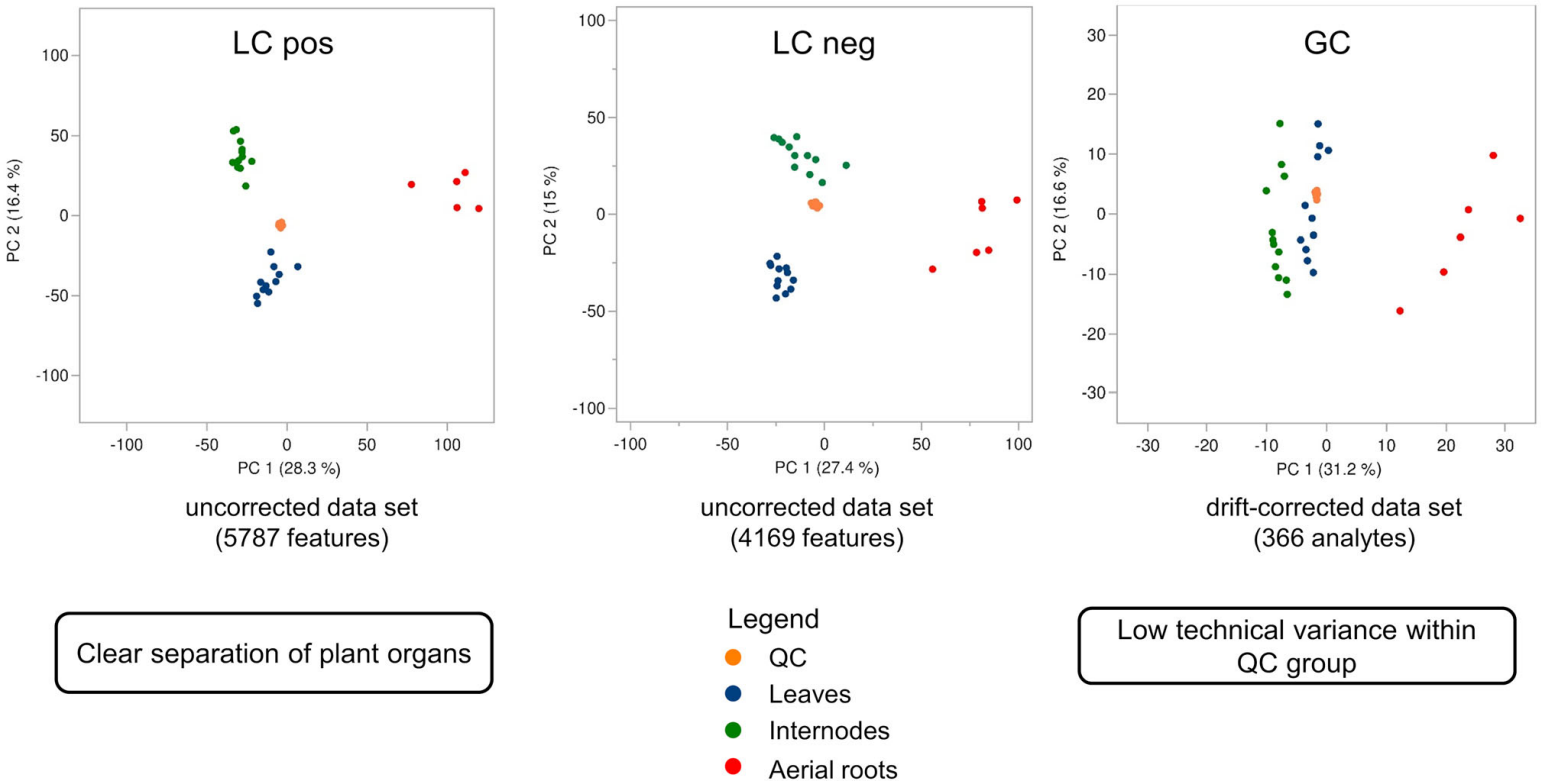
PCA score plots of samples of *Vanilla planifolia* analyzed by three different analytical techniques: LC–MS analysis with positive ionization (left), LC–MS analysis with negative ionization (in the middle), and GC × GC–MS analysis (right). To distinguish the samples from different plant organs, they were colored as follows: QC (orange dots), leaves (blue dots), internodes (green dots), and aerial roots (red dots).

LC–MS data were processed by MS‐DIAL according to the parameter settings given in section 2.4.4. For subsequent multivariate statistics, only valid features were used. Thus, features present in the blanks or not consistently detected in the sample groups were eliminated, as features with high missing rates (>25%) and/or high CV values (>30%) in the QC group. PCA was performed based on the data of the adjusted feature lists to verify whether the QC samples cluster together in the score plots. With respect to the PCA score plots of LC–MS measurements, the QC samples (orange dots) were very close together in both, the data of negative and positive ionization modes, indicating the variance to be very small (Figure 1). To confirm that there were no relevant drift effects a signal drift correction by LOWESS algorithm was performed (see section 2.4.5). By evaluating the peak intensity plots of 100 randomly selected features intensity drift was neither observed for the drift‐corrected nor the uncorrected datasets. In addition, both datasets did not show remarkable differences with respect to the distribution of CV values calculated based on IS or on the entire feature list. Due to these results, the non‐drift‐corrected LC–MS data were used for subsequent statistical analysis (see section 3.3).

The GC × GC–MS dataset consisted of 899 raw analytes overall. For 591 analytes, a signal intensity drift correction could be performed (“Correctables”) because they were detected in at least nine out of 10 QC samples in the batch. The signal intensities of the remaining 308 analytes were not corrected (“Uncorrectables”). This was usually not a problem as the measurement consisted of just one batch and stronger inter‐day drift and offset effects did not take place. In the first step, the 899 raw analytes were then filtered to remove all variables with a CV of >30% in the QC samples (before drift correction). The remaining variables were manually checked further to sort out IS, retention index markers, artifacts, and coeluting analytes. Next, in analogy to the LC–MS dataset, analytes that were detected i) in <75% of the QC samples or ii) not in at least 75% of the samples of at least one plant organ were excluded. According to these rules, 533 out of the 899 raw analytes were discarded and 366 genuine and reliable analytes were used for further statistical analysis. Eighteen analytes were exceptionally accepted because they were qualitatively of interest, e.g., analytes only detected in aerial root samples but not in the QC samples or known analytes with a borderline CV in the QC samples.

### 3.2 Characterization of the metabolome of *V. planifolia*

#### 3.2.1 Number of identified metabolites and compound classes

Prepared vanilla samples (leaves, internodes, aerial roots) were measured using two analytical platforms (UHPLC‐QToF‐MS and GCxGC‐MS). In the case of UHPLC‐QToF‐MS samples were analyzed in negative and positive ionization mode. The first step in data analysis was the identification of metabolites in the vanilla samples. Subsequently, these results are presented and discussed. Organ‐specific differences with respect to the metabolite profiles are addressed in section 3.3.

The used multi‐method approach supported comprehensive characterization of the metabolome.^8^, ^9^ Additionally, samples were prepared under mild conditions (methanolic extraction at 35°C) preventing analyte degradation. Metabolite identification is still regarded as one major bottleneck in LC/MS metabolomics. Based on a published meta‐analysis, it was estimated that only about 20% of papers published in 2020 that were related to LC/MS metabolomics, achieved complete (level 1) metabolite identification.^19^ In another recent study, authors raised concerns about the reliability of the published LC/MS metabolomic data because gross errors and contradictions regarding metabolite identification were found within many papers, such as illogical retention times, contradictory elution order, or implausible results with regard to biology.^20^ In this context, we aimed to perform complete identification (level 1*) for reported metabolites in all possible cases by at least one analytical platform, thus facilitating data interpretation and later use of the data by other researchers. In a few cases, we reported metabolites with confidence level 2 (Table 1 and Supporting Information Table S2), i.e. their structures could not be assigned with certainty either due to coeluting isobars/isomers or because no reference compounds were available. In these cases, metabolites were exclusively identified by means of curated external databases (NIST17 and Fiehn libraries).

**TABLE 1:** Identified metabolites in *Vanilla planifolia* and observed differences between the different plant organs (leaves, aerial roots, and internodes). MS2 score derived by the software Sciex OS. AR, aerial roots; conf level, confidence level (see section 2.6.1); inter, internodes; L, leaves; n.d., not detected; RI, retention time index; RT, retention time; Sim, spectrum similarity.

Compound name	Compound class	Significant differences observed between plant organs	GC×GC–MS			LC–MS (ESI+)			LC–MS (ESI‐)		
			Conf level	RI	Sim	Conf level	RT [min]	MS2 Score	Conf level	RT [min]	MS2 Score
glycerol	alcohols	AR > L = Inter	1*	942	96	3	‐	‐	n.d.	‐	‐
3,4‐dihydroxybenzyl alcohol (protocatechuic alcohol)	alcohols (benzyl alcohols & phenols)	AR > > L (n.d.) = Inter (n.d.)	2	1,370	89	n.d.	‐	‐	n.d.	‐	‐
p‐cresol	alcohols (benzyl alcohols & phenols)	Inter > L (n.d.); AR = L (n.d.) /Inter	1*	822	91	n.d.	‐	‐	n.d.	‐	‐
p‐hydroxybenzyl alcohol	alcohols (benzyl alcohols & phenols)	AR > > L; Inter > L; Inter = AR	2	1,177	91	3	‐	‐	n.d.	‐	‐
*N*‐trans‐feruloyltyramine	amines and amides (amides)	AR > > L = Inter	n.d.	‐	‐	1*	7.02	98.7	1*	7.02	98.7
*N*‐trans‐p‐coumaroyltyramine	amines and amides (amides)	AR > > L = Inter	n.d.	‐	‐	3	‐	‐	1*	6.86	98.5
tyramine	amines and amides (amides)	not significant	1*	1,579	89	n.d.	‐	‐	n.d.	‐	‐
ethanolamine	amines and amides (amino alcohols)	not significant	1*	929	96	n.d.	‐	‐	n.d.	‐	‐
*O*‐phosphoryl‐ethanolamine	amines and amides (amino alcohols)	L > > AR; Inter > AR; L = Inter	1*	1,457	91	n.d.	‐	‐	n.d.	‐	‐
spermidine	amines and amides (polyamines)	L > > AR (n.d.); Inter = L/AR (n.d.)	1*	1,930	91	n.d.	‐	‐	n.d.	‐	‐
acetylcarnitine	amines and amides (quaternary ammonium compounds)	not significant	n.d.	‐	‐	1*	1.08	95.2	n.d.	‐	‐
betaine	amines and amides (quaternary ammonium compounds)	AR > > L = Inter	n.d.	‐	‐	1*	0.99	100.0	n.d.	‐	‐
choline	amines and amides (quaternary ammonium compounds)	AR > L = Inter	n.d.	‐	‐	1*	0.95	100.0	n.d.	‐	‐
proline betaine	amines and amides (quaternary ammonium compounds)	AR > > L = Inter	n.d.	‐	‐	1*	1.07	98.0	n.d.	‐	‐
isomer/isobar of γ‐aminobutyric acid	amino acids	not significant	n.d.	‐	‐	2	0.98	mixed spectrum with choline	3	‐	‐
alanine	amino acids	Inter = AR > L	1*	773	93	n.d.	‐	‐	3	‐	‐
asparagine	amino acids	not significant	1*	1,347	94	1*	0.95	100.0	3	‐	‐
aspartic acid	amino acids	L = Inter > AR	1*	1,200	93	n.d.	‐	‐	3	‐	‐
β‐alanine	amino acids	not significant	1*	1,099	92	n.d.	‐	‐	3	‐	‐
γ‐aminobutyric acid	amino acids	Inter > L; AR = L/Inter	1*	1,203	97	n.d.	‐	‐	3	‐	‐
glutamine	amino acids	Inter > > AR; L = Inter/AR or not significant (similar trend)	1*	1,452	95	1*	0.95	100.0	n.d.	‐	‐
histidine	amino acids	Inter > > L = AR (n.d.)	1*	1,591	91	3	‐	‐	3	‐	‐
isoleucine	amino acids	not significant	1*	962	96	1*	1.66	99.7 (Ile) 100.0 (*allo*‐Ile)	n.d.	‐	‐
leucine	amino acids	AR > > L; Inter = L/AR	1*	937	96	n.d.	‐	‐	n.d.	‐	‐
L‐glutamic‐acid	amino acids	L = Inter > AR or L > Inter > AR (similar trend)	1*	1,301	94	1*	0.97	100.0	3	‐	‐
L‐pipecolic‐acid	amino acids	AR > L; Inter = L/AR	n.d.	‐	‐	1*	1.08	98.7	n.d.	‐	‐
L‐tryptophan	amino acids	Inter > L = AR	1*	1886	96	1*	3.98	99.5	3	‐	‐
methionine	amino acids	not significant	1*	1,191	93	1*	1.51	71.8	n.d.	‐	‐
ornithine	amino acids	not significant	1*	1,493	92	n.d.	‐	‐	n.d.	‐	‐
oxoproline	amino acids	L > AR; Inter = L/AR	1*	1,193	96	n.d.	‐	‐	3	‐	‐
pantothenic acid	amino acids	AR > L = Inter or not significant (similar trend)	1*	1,666	91	1*	3.59	96.3	n.d.	‐	‐
phenylalanine	amino acids	Inter > > L = AR	1*	1,297	94	1*	3.26	100.0	3	‐	‐
proline	amino acids	not significant	1*	965	96	3	‐	‐	n.d.	‐	‐
serine	amino acids	AR > Inter; L = Inter/AR	1*	1,036	94	n.d.	‐	‐	3	‐	‐
threonine	amino acids	AR > L = Inter	1*	964	77	3	‐	‐	3	‐	‐
tyrosine	amino acids	Inter > > L/AR; L > AR or Inter > > AR; L > AR; L = Inter or Inter > > L = AR (similar trends)	1*	1,611	94	1*	1.62	100.0	1*	1.62	95.9
valine	amino acids	not significant	1*	877	96	1*	1.08	96.8	n.d.	‐	‐
α‐linolenic acid	fatty acids and derivatives (fatty acids)	L > Inter = AR	2	1,893	93	n.d.	‐	‐	n.d.	‐	‐
arachidic acid	fatty acids and derivatives (fatty acids)	AR > > L = Inter	2	2,114	92	n.d.	‐	‐	n.d.	‐	‐
behenic acid	fatty acids and derivatives (fatty acids)	AR > > L = Inter	2	2,311	91	n.d.	‐	‐	n.d.	‐	‐
heneicosanoic acid	fatty acids and derivatives (fatty acids)	AR > > L = Inter	2	2,207	88	n.d.	‐	‐	n.d.	‐	‐
hexacosanoic acid	fatty acids and derivatives (fatty acids)	Inter > AR; L = Inter/AR	2	2,700	85	n.d.	‐	‐	n.d.	‐	‐
linoleic acid	fatty acids and derivatives (fatty acids)	AR > Inter; L = Inter/AR	2	1,888	93	n.d.	‐	‐	n.d.	‐	‐
nonadecanoic acid	fatty acids and derivatives (fatty acids)	AR > L = Inter	1*	2,015	93	n.d.	‐	‐	n.d.	‐	‐
octacosanoic acid	fatty acids and derivatives (fatty acids)	L > Inter > AR	2	2,897	91	n.d.	‐	‐	n.d.	‐	‐
pentadecanoic acid	fatty acids and derivatives (fatty acids)	AR > L = Inter	1*	1,622	93	n.d.	‐	‐	3	‐	‐
tetracosanoic acid	fatty acids and derivatives (fatty acids)	AR > L = Inter	2	2,506	93	n.d.	‐	‐	n.d.	‐	‐
triacontanoic acid	fatty acids and derivatives (fatty acids)	L > > AR; L > Inter; Inter > AR	2	3,092	88	n.d.	‐	‐	n.d.	‐	‐
tricosanoic acid	fatty acids and derivatives (fatty acids)	AR > L = Inter	1*	2,408	90	n.d.	‐	‐	n.d.	‐	‐
1‐docosanol	fatty acids and derivatives (fatty alcohols)	AR > > L = Inter	1*	2,222	91	n.d.	‐	‐	n.d.	‐	‐
dotriacontanol	fatty acids and derivatives (fatty alcohols)	L = Inter > > AR	2	3,185	82	n.d.	‐	‐	n.d.	‐	‐
2‐tetracosanoylglycerol	fatty acids and derivatives (fatty esters)	AR > > L = Inter	2	2,990	84	n.d.	‐	‐	n.d.	‐	‐
docosanoylglycerol	fatty acids and derivatives (fatty esters)	AR > > Inter (n.d.) > L (n.d.)	2	2,834	85	n.d.	‐	‐	n.d.	‐	‐
eicosanoylglycerol	fatty acids and derivatives (fatty esters)	AR > > L (n.d.) = Inter (n.d.)	2	2,640	86	n.d.	‐	‐	n.d.	‐	‐
heneicosanoylglycerol	fatty acids and derivatives (fatty esters)	AR > > L (n.d.) = Inter (n.d.)	2	2,740	86	n.d.	‐	‐	n.d.	‐	‐
tricosanoylglycerol	fatty acids and derivatives (fatty esters)	AR > > L (n.d.) = Inter (n.d.)	2	2,931	84	n.d.	‐	‐	n.d.	‐	‐
diosmetin (and possibly traces of coeluting hispidulin)	flavonoids (flavones)	AR > L = Inter	n.d.	‐	‐	1*	8.13	100.0 (diosmin)20.3 (hispidulin)	1*	8.12	100.0 (diosmin)81.6 (hispidulin)
luteolin	flavonoids (flavones)	AR > L = Inter	n.d.	‐	‐	n.d.	‐	‐	1*	7.26	95.1
sinensetin	flavonoids (flavones)	Inter = AR > > L	n.d.	‐	‐	1*	9.21	92.7	n.d.	‐	‐
apigenin	flavonoids (flavones)	AR > > L; AR > Inter; Inter > L	n.d.	‐	‐	3	‐	‐	1*	7.94	98.9
apigenin 6‐*C*‐glucoside and/or apigenin 8‐*C*‐glucoside	flavonoids (flavones)	not significant or AR > L = Inter (similar trend)	n.d.	‐	‐	1*	5.54	97.1 (6‐*C*‐glc) 69.8 (8‐*C*‐glc)	1*	5.54	95.7 (6‐*C*‐glc) 99.2 (8‐*C*‐glc)
vicenin II	flavonoids (flavones)	AR > > L (n.d.) /Inter; Inter > L (n.d.)	n.d.	‐	‐	1*	4.73	99.3	1*	4.73	100.0
apigenin 7‐*O*‐glucoside	flavonoids (flavones)	Inter > L = AR	n.d.	‐	‐	3	‐	‐	1*	6.10	100.0
diosmetin deoxyhexose‐hexose	flavonoids (flavones)	not significant	n.d.	‐	‐	1*	6.05	68.6 (diosmin) good match (neodiosmin)	3	‐	‐
hispidulin 7‐*O*‐glucoside	flavonoids (flavones)	not significant	n.d.	‐	‐	1*	6.23	100.0	1*	6.22	95.3
penduletin	flavonoids (flavonols)	AR > > L/Inter; Inter > L	n.d.	‐	‐	1*	9.43	61.8	1*	9.42	89.3
3,4‐dihydroxybenzaldehyde	hydroxybenzaldehydes	AR > > L = Inter	n.d.	‐	‐	n.d.	‐	‐	1*	4.58	99.8
4‐hydroxybenzaldehyde	hydroxybenzaldehydes	AR > > L = Inter	2	1,164	89	n.d.	‐	‐	1*	5.39	100.0
4‐hydroxybenzaldehyde glucoside	hydroxybenzaldehydes	not significant	n.d.	‐	‐	3	‐	‐	1*	3.83	98.5
glucovanillin	hydroxybenzaldehydes	L > AR > Inter (n.d.)	n.d.	‐	‐	n.d.	‐	‐	1*	4.23	100.0
vanillin	hydroxybenzaldehydes	AR > > L (n.d.) = Inter	2	1,318	88	1*	5.80	100.0	1*	5.80	100.0
arbutin	miscellaneous	not significant	2	2,270	88	3	‐	‐	n.d.	‐	‐
indole‐3‐carbaldehyde	miscellaneous	AR > L = Inter	n.d.	‐	‐	1*	6.50	100.0	3	‐	‐
nicotinic acid	miscellaneous	not significant (L (n.d.); Inter (n.d.))	1*	963	89	n.d.	‐	‐	n.d.	‐	‐
adenine	miscellaneous	not significant or L > AR; Inter = L/AR (different trends for GC and LCpos)	1*	1,535	89	1*	1.08	95.8	n.d.	‐	‐
α‐tocopherol	miscellaneous	L > Inter = AR	2	2,791	80	n.d.	‐	‐	n.d.	‐	‐
δ‐tocopherol	miscellaneous	not significant	2	2,559	82	n.d.	‐	‐	n.d.	‐	‐
phosphate	miscellaneous	Inter > L > AR	1*	937	91	n.d.	‐	‐	n.d.	‐	‐
urea	miscellaneous	not significant	1*	903	96	n.d.	‐	‐	n.d.	‐	‐
2‐isopropylmalic acid	organic acids	AR > Inter > L	2	1,248	85	n.d.	‐	‐	1*	4.35	99.8
3‐hydroxy‐3‐methylbutyric acid	organic acids	not significant	1*	870	88	n.d.	‐	‐	n.d.	‐	‐
adipic acid	organic acids	not significant	1*	1,183	87	n.d.	‐	‐	n.d.	‐	‐
α‐ketoglutaric acid	organic acids	L > Inter > > AR	1*	1,258	98	n.d.	‐	‐	n.d.	‐	‐
citramalic acid	organic acids	not significant	1*	1,144	95	n.d.	‐	‐	n.d.	‐	‐
citric acid	organic acids	L = AR > Inter	1*	1,501	91	n.d.	‐	‐	n.d.	‐	‐
fumaric acid	organic acids	not significant	1*	1,026	94	n.d.	‐	‐	3	‐	‐
homocitric acid	organic acids	L > Inter = AR	n.d.	‐	‐	3	‐	‐	1*	1.58	100.0
maleic acid	organic acids	L = Inter > AR	2	978	93	n.d.	‐	‐	n.d.	‐	‐
malic acid	organic acids	L = Inter > AR	1*	1,167	97	n.d.	‐	‐	n.d.	‐	‐
pyruvic acid	organic acids	not significant	1*	717	95	n.d.	‐	‐	3	‐	‐
suberic‐acid	organic acids	AR > L = Inter	n.d.	‐	‐	n.d.	‐	‐	1*	5.61	97.4
succinic acid	organic acids	not significant	1*	990	97	n.d.	‐	‐	3	‐	‐
3,4‐dihydroxybenzoic acid	phenolic acids (hydroxybenzoic acids)	AR > Inter (n.d.); L (n.d.) = Inter (n.d.) /AR	n.d.	‐	‐	n.d.	‐	‐	1*	3.88	98.2
4‐hydroxybenzoic acid	phenolic acids (hydroxybenzoic acids)	AR > > L = Inter	1*	1,301	94	n.d.	‐	‐	n.d.	‐	‐
vanillic acid	phenolic acids (hydroxybenzoic acids)	AR > > L (n.d.) = Inter	1*	1,438	88	n.d.	‐	‐	1*	4.99	88.4
vanillic acid 4‐*O*‐glucoside	phenolic acids (hydroxybenzoic acids)	Inter > L > AR or Inter > L = AR (similar trend)	n.d.	‐	‐	1*	3.59	99.2	1*	3.59	100.0
3,5‐dimethoxy‐4‐hydroxycinnamic‐acid	phenolic acids (hydroxycinnamic acids)	L > Inter = AR	1*	1,924	89	n.d.	‐	‐	1*	5.91	99.3
caffeic‐acid	phenolic acids (hydroxycinnamic acids)	AR > L = Inter	n.d.	‐	‐	3	‐	‐	1*	4.92	98.8
ferulic acid	phenolic acids (hydroxycinnamic acids)	AR > Inter or AR > L = Inter (similar trend)	1*	1,764	89	n.d.	‐	‐	1*	5.93	98.9
ferulic acid 4‐*O*‐glucoside	phenolic acids (hydroxycinnamic acids)	L > Inter = AR	n.d.	‐	‐	1*	4.26	100.0	3	‐	‐
p‐coumaric acid	phenolic acids (hydroxycinnamic acids)	AR > > L = Inter	1*	1,608	94	n.d.	‐	‐	1*	5.64	100.0
*N*‐acetylglucosamine	sugars (amino sugars)	AR > L; Inter = L/AR	1*	1,756	92	n.d.	‐	‐	n.d.	‐	‐
arabinose	sugars (monosaccharides)	AR > L; Inter = L/AR	1*	1,342	95	n.d.	‐	‐	n.d.	‐	
fructose	sugars (monosaccharides)	Inter > L	1*	1,558 1,568	93 94	3	‐	‐	3	1.00	96.5
glucose	sugars (monosaccharides)	Inter = AR > L	1*	1,579 1,594	94 94	n.d.	‐	‐	n.d.	‐	‐
glyceraldehyde	sugars (monosaccharides)	Inter > > L = AR	2	880	90	n.d.	‐	‐	n.d.	‐	‐
threose	sugars (monosaccharides)	L > > Inter; AR = L = Inter	2	1,116	92	n.d.	‐	‐	n.d.	‐	‐
xylose	sugars (monosaccharides)	AR > L; Inter = L/AR	1*	1,328	93	n.d.	‐	‐	3	‐	‐
β‐gentiobiose	sugars (oligosaccharides)	AR > > L; AR > Inter; Inter > L or AR > L = Inter (similar trend)	1*	2,505	92	n.d.	‐	‐	n.d.	‐	‐
raffinose or other trisaccharide	sugars (oligosaccharides)	L = Inter > AR	2	3,052	82	n.d.	‐	‐	n.d.	‐	‐
sucrose	sugars (oligosaccharides)	L = Inter > AR	1*	2,314	92	3	1.04	100.0	3	‐	‐
3‐deoxyhexonic acid γ‐lactone	sugars (sugar acids)	AR > L = Inter	2	1,471	89	n.d.	‐	‐	n.d.	‐	‐
threonic acid	sugars (sugar acids)	not significant	1*	1,244	95	n.d.	‐	‐	n.d.	‐	‐
arabitol	sugars (sugar alcohols)	AR > > L = Inter (n.d.)	1*	1,405	88	3	‐	‐	n.d.	‐	‐
galactinol	sugars (sugar alcohols)	not significant	1*	2,656	93	n.d.	‐	‐	n.d.	‐	‐
mannitol	sugars (sugar alcohols)	AR > > L/Inter; L > Inter	1*	1,609	93	3	‐	‐	3	‐	‐
meso‐erythritol	sugars (sugar alcohols)	AR > > L = Inter (n.d.)	1*	1,191	96	n.d.	‐	‐	n.d.	‐	‐
myo‐inositol	sugars (sugar alcohols)	not significant	1*	1,763	91	3	‐	‐	3	‐	‐
sorbitol	sugars (sugar alcohols)	AR > L = Inter	1*	1,616	93	3	‐	‐	3	‐	‐
threitol	sugars (sugar alcohols)	L = AR > Inter	1*	1,184	84	n.d.	‐	‐	n.d.	‐	‐
glucose‐6‐phosphate	sugars (sugar phosphates)	Inter > > AR; L = Inter/AR	1*	1,997	85	n.d.	‐	‐	n.d.	‐	‐
glycerol‐1‐phosphate	sugars (sugar phosphates)	L > > AR; L > Inter; Inter > AR	2	1,445	81	n.d.	‐	‐	n.d.	‐	‐
neophytadiene isomer	terpenes/terpenoids(diterpenes)	L > > AR; L > Inter; Inter > AR	2	1,509	94	n.d.	‐	‐	n.d.	‐	‐
phytol	terpenes/terpenoids(diterpenes)	L > Inter > AR	1*	1,853	94	n.d.	‐	‐	n.d.	‐	‐
β‐sitosterol	terpenes/terpenoids(phytosterols)	L = AR > Inter	2	2,981	85	n.d.	‐	‐	n.d.	‐	‐
campesterol	terpenes/terpenoids(phytosterols)	AR > L = Inter	2	2,903	91	n.d.	‐	‐	n.d.	‐	‐
stigmasterol	terpenes/terpenoids(phytosterols)	AR > L = Inter	2	2,927	89	n.d.	‐	‐	n.d.	‐	‐

For metabolite identification in LC–MS analyses an in‐house database was used comprising the retention times and MS2 data of approx. 550 reference compounds were acquired under identical experimental conditions (see Supporting Information Data S6). Regarding annotation using accurate mass and retention time, 106 and 87 features were annotated in negative and positive polarity, respectively. After the exclusion of unreliable results 78 and 64 features were retained for positive and negative polarity, respectively. To enable unequivocal identification, the MS2 spectra acquired were compared with reference spectra from the in‐house database. If different isobars/isomers had to be considered for a feature due to similar retention behavior (ΔRT ≤ 0.13 min), spectral matching was performed for all relevant candidates with the aim to exclude as many of them as possible.

For GC × GC–MS analysis, samples were derivatized by methoximation/silylation, and compounds were chromatographically separated on a two‐dimensional system comprising an apolar x semipolar column combination. In the case of GC × GC–MS analysis 142 analytes were annotated based on retention indices and spectral matching using an in‐house spectral library and the commercially available NIST17 and Fiehn libraries (see section 2.6.1).

**FIGURE 2:**
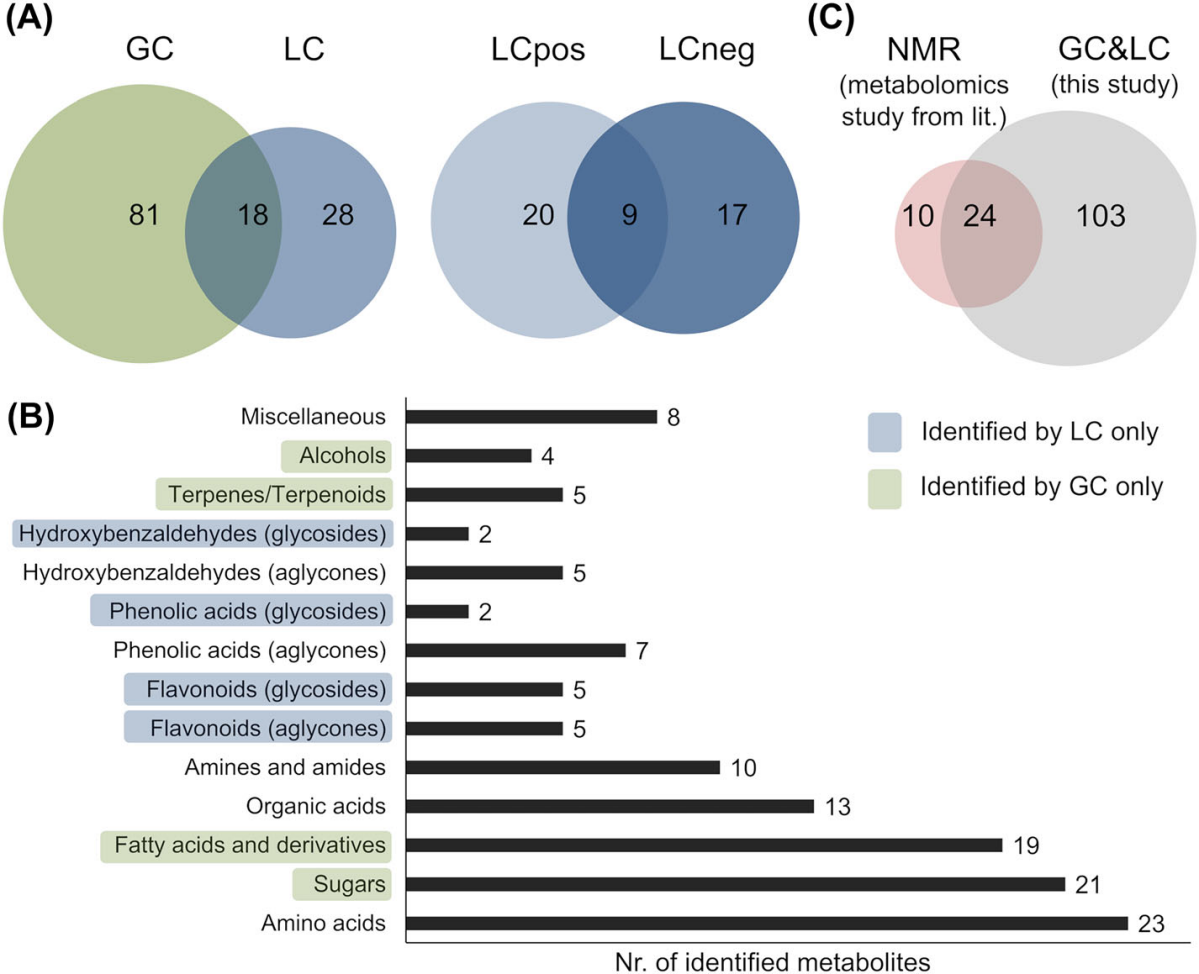
Results of metabolite identification in vanilla plants. Number of metabolites identified by LC and/or GC platform (left and middle) or with respect to LC positive mode and/or LC negative mode (right) displayed as Venn diagrams (A), histogram of assigned compound classes (B), and comparison of the data of this study with the results of an NMR‐based metabolomics study of Leyva et al. (2021)^13^ (C).

After manual curation, 29, 26, and 99 metabolites were reliably identified (level 1* or 2) using LC–MS positive polarity, LC–MS negative polarity, and GC × GC–MS, respectively. Subsequently, the identification result lists of the three analytical techniques were merged into one table (Table 1; Supporting Information Table S2). Considering that some metabolites are detected by more than one platform, we identified 127 distinct metabolites in the vanilla samples in total. By means of GC–MS analysis, 81 metabolites were identified, which were not detected by LC–MS (Figure 2A). On the other hand, 28 metabolites were shown to be LC‐specific. This confirms that the metabolome coverage can be significantly enhanced when using a combination of an LC‐QToF‐MS‐ and GC × GC–MS‐based metabolomic platform. Only nine of 46 metabolites were identified in both positive and negative mode demonstrating the benefit of performing LC–MS measurements in both polarities (Figure 2A). All reported metabolites were identified either based on confidence level 1* or 2. Confidence level 1* was achieved for 96 of 127 metabolites (in total), for 66 of 99 metabolites (GC × GC–MS), and for 45 of 46 metabolites (LC‐QToF‐MS). Sixteen metabolites were identified to be unique, i.e. not detected in all plant organs (Table 1). For details see section 3.3.

Among the number of primary metabolites, amino acids (23) were most prevalent, followed by sugars (21), fatty acids and derivatives (19), organic acids (13), and amines/amides (10) (Figure 2B). In the case of secondary plant metabolites mainly flavonoids (10), phenolic acids (9), hydroxybenzaldehydes (5), and terpenoids (5) were identified. Fatty acids and derivatives, sugar compounds, phenols and terpenes/terpenoids were identified only by GC–MS, whereas flavonoids, some hydroxybenzaldehydes and phenolic acids (caffeic acid, 3,4‐dihydroxybenzoic acid, 3,4‐dihydroxybenzaldehyde), and all glycosides of secondary metabolites were exclusively identified with LC–MS (Table 1). Although the GC–MS‐based metabolomic platform used here was obviously crucial for the comprehensive characterization of the vanilla metabolome, the results also clarified that a relevant number of secondary metabolites would have been overlooked, if no LC–MS analysis had been performed.

To the best of our knowledge, there was only one study known from the literature characterizing the leaf metabolome of *V. planifolia* by metabolomics approach.^13^ In this study, 36 metabolites (or 34 if the different isomeric forms of glucose and fructose are not counted) were reported based on NMR analysis. Thereof 24 metabolites could be confirmed in our study (Figure 2C) including 12 amino acids (alanine, valine, leucine, isoleucine, γ‐aminobutyric acid, glutamine, asparagine, aspartic acid, threonine, tyrosine, phenylalanine, proline), six organic acids (fumaric acid, succinic acid, citric acid, homocitric acid, malic acid, pyruvic acid), three sugars (glucose, fructose, and sucrose), choline, vanillic acid, and p‐hydroxybenzyl alcohol. In contrast to Leyva et al^13^ where proline and pyruvic acid were not detected in leaves, both metabolites could be reliably identified in leaf samples of our study (Table 1). This could be explained by lower sensitivity in NMR analysis. On the other hand, 10 metabolites previously reported by Leyva et al^13^ (arginine, acetic acid, ethanol, glycine, glucoside A, glucoside B, 4‐hydroxybenzylalcohol glycoside, lactone form of homocitric acid, lactic acid, lysine) could not be identified in our study, probably due to various reasons: (i) poorer detectability with regard to the analytical methods used, (ii) no availability of the respective reference compound, (iii) coelution of analytes that prevented reliable identification, or (iv) potential accession‐related differences.

#### 3.2.2 Phenolic acids, phenols, hydroxybenzaldehydes (vanillin‐related metabolites)

**FIGURE 3:**
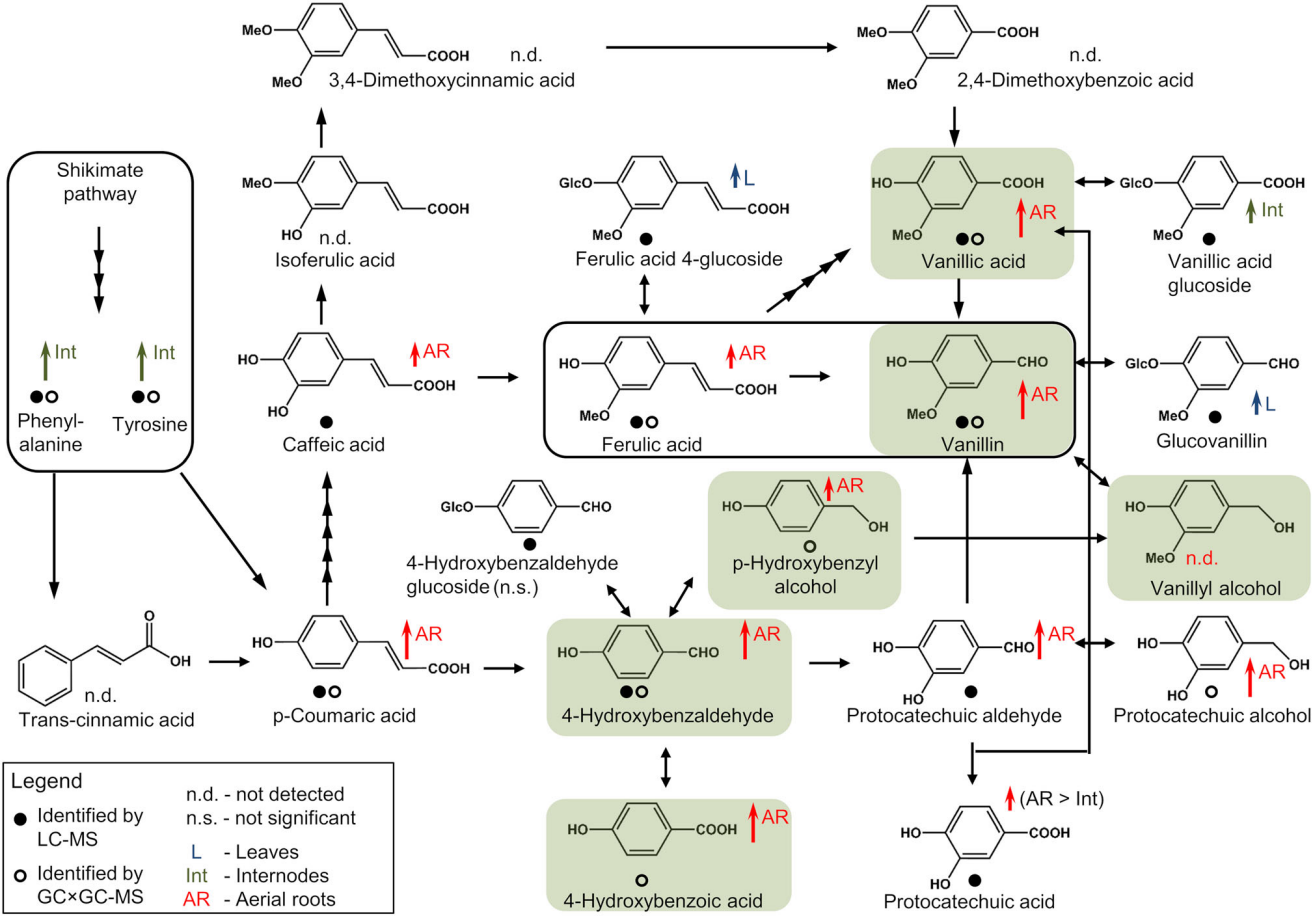
Vanillin biosynthesis in *Vanilla planifolia* . The scheme was originally adapted from Kundu (2017)^15^ and slightly modified.^3^, ^21^, ^22^, ^23^, ^24^, ^25^, ^26^ Some given metabolic conversions consisted of more than one reaction step. Therefore, multiple arrows within one line were used indicating one biochemical reaction step for each arrow. Colored arrows indicate the significant differences in the metabolite levels observed between different organs of the vanilla plant. The arrow length reflects the fold‐change (FC) observed: short arrows, FC <5; long arrows, FC ≥5. Metabolites were marked with a filled dot and/or an unfilled dot depending on the detection method (LC–MS and/or GC–MS). The chemical structures of aroma‐active metabolites that contribute to the typical vanilla flavor were highlighted within green boxes. All metabolites were identified based on a comparison with standard reference material.

We identified 31 metabolites (Supporting Information Table S7) that according to the literature^3^, ^15^, ^21^, ^22^, ^23^, ^24^, ^25^, ^26^ are related to vanillin biosynthesis in *V. planifolia*. With the exception of phenylalanine and tyrosine, all those metabolites belonged to phenolic acids, simple phenols, and hydroxybenzaldehydes. In our study, five hydroxycinnamic acids and four hydroxybenzoic acids were identified in the class of phenolic acids in vanilla plants (Table 1). In addition, three simple phenols and five hydroxybenzaldehydes were shown to be present (Table 1). With the exception of sinapic acid (3,5‐dimethoxy‐4‐hydroxycinnamic‐acid), all detected metabolites belonging to these three classes were already proposed as precursors/intermediates in vanillin biosynthesis (Figure 3). Among them, vanillic acid and p‐hydroxybenzylalcohol were shown to occur in *V. planifolia* leaves and stems.^13^ However, in contrast to Funk and Brodelius (1990),^23^, ^24^ trans‐cinnamic acid, isoferulic acid, 3,4‐dimethoxycinnamic acid, and 2,4‐dihydroxybenzoic acid were neither detected with GC nor LC. Thus, the pathway proposed by the authors is not supported by our data because none of the respective intermediates were identified (Figure 3). These results suggest that vanillin is likely formed either from phenylalanine via lignin precursors according to ferulic acid‐mediated pathway or via proposed non‐β‐oxidative pathway, where 4‐hydroxybenzaldehyde and 3,4‐dihydroxybenzaldehyde were discussed as potential intermediates.^21^, ^25^, ^26^ Another conceivable route is the synthesis of glucovanillin from p‐hydroxybenzyl alcohol glucoside.^15^

#### 3.2.3 Flavonoids

**FIGURE 4:**
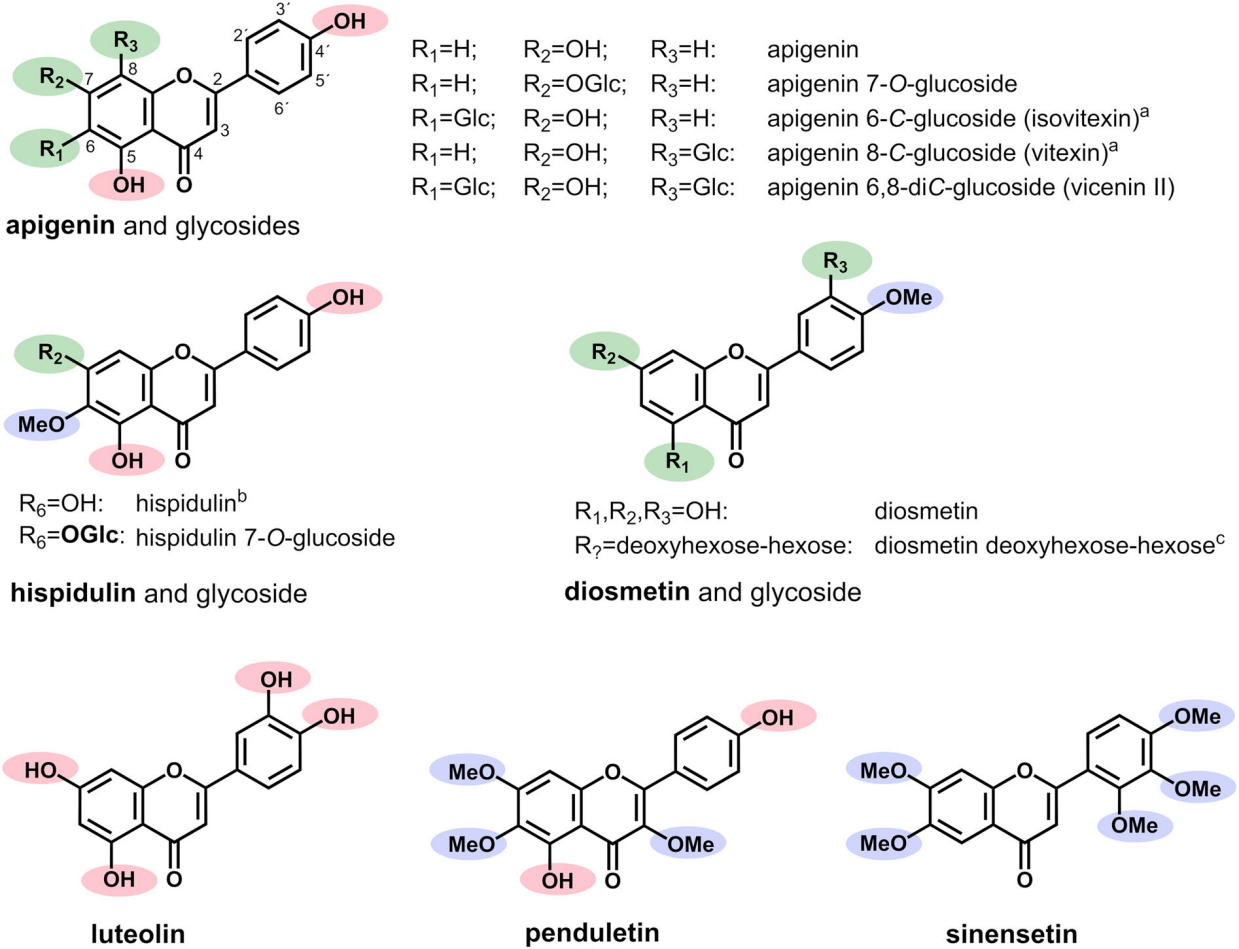
Flavonoids identified in vanilla plants; ^a^apigenin 6‐*C*‐glucoside and apigenin 8‐*C*‐glucoside cannot be clearly distinguished by the LC–MS method used here; ^b^hispidulin may be present as a minor component, but identification is uncertain due to coelution with the more abundant isomer diosmetin. ^c^diosmin and neodiosmin were excluded after spiking experiments.

Several aglycones of flavonoids including apigenin, diosmetin, luteolin, sinensetin, penduletin, and potentially hispidulin were detected (Figure 4). Due to coelution effects, a differentiation of isomeric diosmetin and hispidulin was challenging. While the respective MS2 reference spectra are quite similar in negative mode ([M‐H]^−^), their fragmentation patterns recorded in positive mode ([M + H]^+^) show significant differences. Due to the absence of two characteristic fragments for hispidulin (m/z 168 and m/z 140) in the recorded sample spectrum and the high abundance of a fragment with m/z 258, which was present in both reference spectra, but displaying a clearly higher intensity in the spectrum of diosmetin, it can be concluded that diosmetin must be the major metabolite, and hispidulin is only present as a minor component, if at all. With respect to hispidulin and apigenin, their respective 7‐*O*‐glucosides were also identified. A glycoside could also be detected for diosmin (Table 1). According to our in‐house database, the retention time (6.02 min) and accurate mass (m/z 609.1861) of this glycoside fit well with diosmin (diosmetin 7‐*O*‐neohesperidoside). But even if the acquired spectrum and the reference spectrum show similarities (purity score 68.6), there was one apparent difference. The intense fragment with an m/z of 463.1214 from the reference spectrum formed by cleavage of the deoxy‐hexosyl moiety, was detected in the sample spectrum only as a minor fragment. A spiking experiment revealed a very slight retention time shift. Thus, we assumed the metabolite to be an isomer of diosmin. The comparison with the in‐house spectrum of neodiosmin (diosmetin 7‐*O*‐rutinoside) showed good spectral matching, but retention time deviation (0.05 min) was comparatively high, and by means of a spiking experiment, neodiosmin had to be excluded, too. Thus, the metabolite is supposed to be another deoxyhexose‐hexose conjugate of diosmetin. The detection of apigenin accompanied with at least two *C*‐glucosides, namely apigenin 6,8‐di‐*C*‐glucoside (vicenin II), apigenin 6‐*C*‐glucoside (isovitexin), and/or apigenin 8‐*C*‐glucoside (vitexin). In the case of vitexin and isovitexin a differentiation based on their retention times and MS2 spectra was not possible. While all other flavonoids identified here belong to flavones, penduletin was the only flavonol found (Figure 4).

Busconi et al^10^ analyzed the phenolic profiles of cured pods of *V. x tahitensis* thus annotating 260 phenolic compounds. Next to other classes (phenolic acids, lignans, stilbenes, and other polyphenols), a particularly high number of flavonoid metabolites (120) was reported. But apparently, compound annotation was only performed based on the comparison of accurate mass and retention time. For this reason, the reported metabolites need to be considered with caution and are thus not discussed in detail here. Besides this work, literature data for flavonoids in vanilla plants are scarce. Díaz‐Bautista et al^27^ investigated the presence of secondary plant metabolites in the stem and leaves of different plants belonging to *Vanilla* genus based on chemical derivatization and subsequent analysis by thin‐layer chromatography. Here, flavonoids were not detected in *V. planifolia* but for other species (*Vanilla pompona*, *V. insignis*, *V. indora*). In a review of the phytochemical composition of orchids, the presence of flavonoids metabolites such as quercetin, kaempferol, and isorhamnetin derivatives was mentioned for different orchids, but no *Vanilla* species was among them.^28^ In a recent study, strikingly high amounts of tricin‐lignin were found in aerial roots of *V. planifolia*. Tricin is an *O*‐methylated flavone that was recognized as the first true lignin monomer outside the canonical monolignol biosynthetic pathway, which suggests a possible link between monolignol and flavonoid biosynthesis.^29^, ^30^

Due to their antioxidant properties flavonoids can protect the plant against infection by pathogenic fungi and bacteria. Substitution with hydroxyl and methoxy groups has been shown to generally reduce antioxidant capacity, although for some methylated flavonoids higher antioxidant effects have been observed.^31^ In another study, it was demonstrated that antifungal activity against *Fusarium* tends to be higher for flavones as compared to flavanones.^32^ With vitexin/isovitexin, two flavones detected in this study, were reported to be effective against pathogenic *Fusarium oxysporum*,^33^ which can cause serious crop disease in *V. planifolia*.^34^

#### 3.2.4 Terpenoids/terpenes

Phytosterols are a normal constituent of the plasma membrane of plant cells and their occurrence in the outer wax layers of plant leaves is also well‐known.^35^, ^36^ Furthermore, they have been related to responses against abiotic and biotic stress.^37^ Three different phytosterols, namely β‐sitosterol, stigmasterol, and campesterol, were detected that belong to the most commonly found representatives in plants.^38^ Next to phytosterols, two diterpenes (phytol, neophytadiene) were identified. So far, hardly any literature data is available on the occurrence of terpenes in vanilla. In relation to orchids, a review from 2010 described a few terpenoids, none of which were associated with vanilla.^28^ To the best of our knowledge, no phytosterols have been described for *V. planifolia* so far. Apparently, there is one study showing the presence of phytosterols and diterpenoids in *V. borneensis* (a rare endemic climbing orchid from India) based on a qualitative phytochemical screening, but no distinct metabolites were identified in this study.^39^

#### 3.2.5 Fatty acids and derivatives

The detected fatty acids contained ten saturated fatty acids with a chain length ranging from C15 (pentadecanoic acid) to C30 (triacontanoic acid) and the two unsaturated metabolites, namely α‐linolenic acid (18:3ω3) and linoleic acid (18:2ω6). In addition, five glyceroyl esters were identified (Table 1). Brunschwig et al^4^ studied the fatty acid profile of *V. planifolia* beans. Nine fatty acids (C16 to C30) were quantified by means of GC–MS including α‐linolenic acid (18:3ω3) and linoleic acid (18:2ω6) with the latter one found to be the most abundant metabolite (46–64% related to the relative fatty acid composition of the analyzed fatty acids). Based on GC × GC–MS data, linoleic acid also belonged to the fatty acids with the highest intensities. However, in contrast to Brunschwig et al,^4^ where α‐linoleic acid only accounts for a minor part of total fatty acid composition (1.9–5.4%), here, α‐linoleic acid was found in higher quantities than linoleic acid (data not shown). While methyl and ethyl ester derivatives of fatty acids including the previously mentioned ones have already been reported in literature,^40^, ^41^ this is the first time that the presence of glycerol‐derived fatty acid esters has been demonstrated for vanilla. Noteworthy, the ester bonds of lipids can, at last partially, be cleaved upon incubation with silylation reagents like MSTFA. It is therefore not entirely clear to which extent the aforementioned fatty acids and glyceroyl esters occurred in the plants freely or were liberated from, e.g., phospholipids or wax esters.

#### 3.2.6 Sugars

In the class of sugars four monosaccharides (glucose, fructose, threose, and xylose), three oligosaccharides (sucrose, β‐gentiobiose, raffinose, or another trisaccharide), and a large number of sugar derivatives were detected (Table 1) comprising seven sugar alcohols (e.g. mannitol, sorbitol, galactinol), two sugar acids (e.g. threonic acid and 3‐deoxyhexonic acid γ‐lactone), glucose 6‐phosphate and glycerol 1‐phosphate as well as *N*‐acetylglucosamine. From the sugars identified in this study, only glucose, fructose, and sucrose were described to occur in leaves and/or stems of *V. planifolia* so far.^13^ All sugars were identified by GC × GC–MS allowing good separation of existing sugar isomers. Because of the high separation performance of this platform, many more sugars or sugar‐related compounds could be tentatively identified (level 3; data not shown) due to the presence of characteristic sugar fragments in their mass spectra. Especially striking was the occurrence of a vast number of sugar‐like compounds in the aerial root samples. These analytes were mainly located before and around the “disaccharide region” of the two‐dimensional chromatogram and often exhibited specific fragments not common for simple sugars as well, suggesting that many of them could be glycosides or other sugar conjugates. Due to a lack of suitable reference compounds and the high spectral similarity of these compounds, structural elucidation was mostly not feasible so far. Regarding LC–MS analysis, identification of single sugar molecules was not possible due to the use of a C18 column leading to the coelution of sugars in the injection peak.

#### 3.2.7 Amines and amides

Four quaternary ammonium compounds (choline, betaine, proline betaine, acetylcarnitine) were detected, of which only choline was already described to be present in leaves/stems of *V. planifolia*.^13^ Further identified metabolites were spermidine, ethanolamine, and its *O*‐phosphoryl derivative as well as tyramine, the biogenic amine derived from tyrosine, which was detected in its free form and as amide conjugate with ferulic acid and p‐coumaric acid, respectively. As with the fatty acids, ethanolamine and *O*‐phosphorylethanolamine could be present in the plant material freely or as part of phosphatidylethanolamines.

#### 3.2.8 Organic acids

In the case of organic acids, well‐known plant metabolites such as representatives of the citric acid cycle (citrate, α‐ketoglutarate, succinate, fumarate, and malic acid) and intermediates of leucine biosynthesis (isopropylmalic acid, 3‐hydroxy‐3‐methylbutyric acid) were found (Table 1). Five of them have already been found in leaves and/or stems of *V. planifolia* including fumaric acid, succinic acid, citric acid, homocitric acid, and malic acid. Pyruvic acid was identified in other *Vanilla* species.^13^ Dicarboxylic acids such as adipic acid or suberic acid were also detected in our studies, and those have been identified as plant metabolites, e.g. in *Arabidopsis*.^42^

#### 3.2.9 Amino acids and vitamins

The detectable amino acid profile (including derivatives) comprised 23 metabolites, thereof 12 metabolites that have already been detected in vanilla leaves and/or steams by Leyva et al^13^ including alanine, valine, leucine, isoleucine, γ‐aminobutyric acid, glutamine, asparagine, aspartic acid, threonine, proline, tyrosine, phenylalanine. But, in contrast to our findings, proline was not detected in *V. planifolia*, but in *V. ribeiroi*. Moreover, 11 additional amino acids or derivatives were identified in our study, i.e. β‐alanine, glutamic acid, histidine, methionine, ornithine, oxoproline, pantothenic acid (vitamin B5), pipecolic acid, serine, tryptophan, and an unknown isomer of γ‐aminobutyric acid (Table 1). In leaves and internodes, the aromatic amino acids phenylalanine, tyrosine and tryptophan were among the top 10 metabolites with respect to their intensities (based on LC–MS data). Aromatic amino acids are synthesized via the shikimate pathway and play a crucial role in plant survival and adaptability, e.g. they are well‐known to be involved in the synthesis of a variety of secondary metabolites, such as the production of phenolic acids and flavonoids via the phenylpropanoid pathway.^43^

Next to pantothenic acid (vitamin B5), an amino acid derivative formed from β‐alanine, three further vitamins were identified including nicotinic acid (vitamin B3), α‐tocopherol and δ‐tocopherol (vitamin E) (Table 1).

### 3.3 Statistically significant differences in metabolite patterns between leaves, internodes, and aerial roots

In order to obtain an overview of the potential distinctness of plant organs at the metabolome level, PCA was performed. From the respective score plots (Figure 1) it can be recognized that the samples originating from different plant organs are well separated from each other. The aerial root samples differed particularly clearly from the samples of the other plant organs. Regarding LC–MS data, the separation of aerial root samples was mainly based on principal component (PC) 1, while leaves and internodes were primarily separated by means of PC 2. In general, the differentiation of plant organs is even more pronounced in the plots obtained from LC–MS data as compared to GC–MS, which may be explained by the differences in the coverage of compound classes by these platforms. Based on the results of multivariate statistics, a high number of significantly different metabolites was expected. To prove this hypothesis, univariate statistics were performed for all metabolites identified (see section 2.6.2). First, a Kruskal‐Wallis test was applied to test for significant differences between all groups (plant organs). Subsequently, the non‐parametric Steel Dwass post‐hoc test was performed in order to test for significance between the distinct group pairs (leaves/internodes, leaves/aerial roots, or internodes/aerial roots). Statistical results are summarized in Table 1. As expected from the PCA score plots (Figure 1) significant differences between the plant organs were observed in the case of 98 metabolites. Subsequently, the results of univariate statistical analysis are presented and discussed for each compound class.

#### 3.3.1 Vanillin‐related metabolites

In general, metabolites related to vanillin biosynthesis (Figure 3) tended to be highly present in aerial roots (10 out of 17 metabolites). Vanillin, vanillic acid, and 3,4‐dihydroxybenzyl alcohol (protocatechuic alcohol) were not detected in leaves (Table 1). The high abundance of vanillin‐related metabolites in aerial roots was most obvious (fold changes >5) for p‐coumaric acid, hydroxybenzaldehydes (vanillin, 4‐hydroxybenzaldehyde, 3,4‐dihydroxybenzaldehyde), hydroxybenzoic acids (4‐hydroxybenzoic acid and vanillic acid), and hydroxybenzyl alcohols (p‐hydroxybenzyl alcohol, 3,4‐dihydroxybenzylalcohol). Ferulic acid and caffeic acid showed similar effects as p‐coumaric acid but less pronounced. While in the case of p‐hydroxybenzyl alcohol, significantly higher intensities in aerial roots were only found with respect to leaf samples, for all other metabolites mentioned above significant differences were observed with respect to leaves and internodes. 3,4‐dihydroxybenzoic acid (protocatechuic acid) and 3,4‐dihydroxybenzylalcohol (protocatechuic alcohol) were identified to be the only vanillin‐related metabolites exclusively detected in aerial roots.

A key question related to the higher occurrence of vanillin and other aldehydes in aerial roots is whether these metabolites are biosynthesized or are mainly stored in this plant organ. It is known that aerial roots can exhibit biosynthetic activity including photosynthesis.^44^ However, this question cannot be finally elucidated by the current study and has to be addressed in further investigations. A reason for the higher intensities of vanillin (and related aldehydes) and of phenolic acids (e.g. vanillic acid) in aerial roots might be their role in defense against pathogens.^45^ In general, roots represent an opportunistic entryway for pathogens.^46^ However, plants can change the composition of the rhizosphere and protect themselves against pathogens by the release of high and low molecular compounds from the roots including flavonoids and hydroxycinnamic acids.^47^ Moreover, root‐derived metabolites such as terpenoid aldehydes and alkaloids have been shown to be important for the defense against leaf attackers.^48^ Roots thus play an important role in pathogen defense and this may also explain the higher intensities of vanillin (and related aldehydes) and phenolic acids in aerial roots. This interpretation is strengthened by the fact that higher levels of indole‐3‐carbaldehyde were found in aerial roots (Table 1), as indolic secondary metabolites are supposed to be involved in pathogen defense, too.^49^ However, it remains unclear whether the mechanisms described for soil roots also apply to aerial roots and therefore these interpretations need to be tested in further studies.

It is also noteworthy that all glucosides associated with the biosynthesis of vanillin are highly abundant in leaves and/or internodes. In the case of glucovanillin increased levels were observed in leaves, while it was not detected in internodes. No significant effects or comparable trends were observed for arbutin, a hydroquinone glucoside formed from p‐coumaric acid via 4‐hydroxybenzoic acid. In general, glycosylation enhances transportation efficiency and glycosides can serve as a storage form of aglycones in the leaves.^50^

#### 3.3.2 Flavonoids

For most of the identified flavonoids (6 out of 10), the highest levels were detected in aerial roots. This was true for the aglycone metabolites diosmetin, luteolin, penduletin, and apigenin, as well as for apigenin *C*‐glycosides (Figure 4). For apigenin 6‐*C*−/−8‐*C*‐glucoside, significance was reached only in positive polarity (aerial roots > leaves = internodes), but results in negative polarity showed a similar trend (data not shown). Very large differences (> 5) were observed for penduletin, apigenin, and vicenin II, although the latter could not be detected in the leaves. In contrast, flavonoid *O*‐glycosides showed a different behavior. Hispidulin 7‐*O*‐glucoside and diosmetin‐deoxyhexose‐hexose showed higher levels in leaves, but these were not significant due to high group variance (data not shown). Apigenin 7‐*O* glucoside was increased in internodes and exhibited lower intensities in leaves and aerial roots (Table 2). In summary, flavonoids showed a comparable pattern in different organs as already observed for vanillin‐related aldehydes. For many aglycones higher intensities were detected in aerial roots, whereas *O*‐glycosides exhibited higher levels in leaves or internodes. Flavonoids also play an important role in plant defense^31^, ^33^, ^50^ and the increase of flavonoid aglycones in aerial roots could be explained in a similar way as for vanillin‐related aldehydes (see section 3.3.1).

#### 3.3.3 Terpenoids/terpenes

Sub‐class‐specific effects occurred within the class of terpenoids/terpenes. While the diterpenes (phytol and neophytadiene) showed the highest intensities in leaves, high phytosterol levels were found in aerial roots. Metabolomic analysis of different organs of the orchid *Dendrobium catenatum* revealed tissue‐specific accumulation of terpenes, including triterpenes in the roots, monoterpenes in the flowers, and sesquiterpenes in the stems.^51^

#### 3.3.4 Fatty acids and derivatives

For most fatty acids (14 of 19) and their derived esters and alcohols, the highest intensities were observed in aerial roots (Table 1), whereas the glyceroyl esters of docosanoic acid, eicosanoic acid, henicosanoic acid, and tricosanoic acid were not detected in leaves and internodes at all. In contrast to this finding, fatty acid derivatives with 26 or more carbon atoms were significantly increased in leaves. This seems plausible for two reasons: first, long‐chain fatty acids are typical constituents of the cuticular wax layer on leaves,^52^ and second, they are involved in organ morphogenesis,^53^ which fits well with the fact, that the vanilla plants investigated here were in the vegetative phase of growth. Also, α‐linolenic acid, which is the starting point for jasmonate synthesis in plants,^54^ was increased in leaves. Jasmonate itself is an important signaling molecule that controls, among other things, plant defense and growth.

#### 3.3.5 Sugars

The distribution of sugars between the plant organs showed a different picture. Four sugar alcohols (arabitol, meso‐erythritol, mannitol, and sorbitol) showed significantly higher intensities in aerial roots (Table 1), even though the first two of them were not detected in internodes. C6 sugar alcohols may serve as carbohydrate reserves.^55^ Additionally, it is well‐described that roots can be a site of carbohydrate storage,^56^, ^57^, ^58^ which could explain the higher levels of mannitol and sorbitol in aerial roots. On the other hand, they are osmoprotective compounds that accumulate in plants under abiotic stress conditions.^59^

Intensities of both, glucose and fructose, were significantly lowered in leaves (Table 1). This likely reflects the direct biosynthesis of oligosaccharides from these monosaccharides, which is in accordance with the observation that sucrose and raffinose revealed elevated levels in leaves and internodes. In most plants, sucrose is the main transport form of photosynthetically produced sugars,^56^, ^57^ but in *V. planifolia* starch is the major storage carbohydrate.^60^ Raffinose is a sugar metabolite typically found in plants that is produced from sucrose and activated galactinol moieties.^61^ Galactinol was also detected, but its levels did not show any significant differences between the plant organs. However, in contrast to sucrose, β‐gentiobiose showed lower intensities in leaves but was higher in aerial roots (Table 1).

#### 3.3.6 Amines and amides

Quaternary ammonium compounds tended to exhibit higher intensities in aerial roots (choline, betaine, proline betaine, and acetylcarnitine) suggesting that aerial roots may have been exposed to higher stress.^59^ Other amines were either unchanged (tyramine and ethanolamine) or had elevated levels in the leaf samples, as in the case of spermidine and *O*‐phosphorylethanolamine. While spermidine was the only compound of this class that was not detected in aerial roots, the amide conjugates of p‐coumaric acid and ferulic acid showed high abundance. This fits well with the observation that the corresponding phenolic acids behaved in the same way (see section 3.3.1).

#### 3.3.7 Organic acids

The levels of organic acids either showed no significant differences (3‐hydroxy‐3‐methylbutyric acid, adipic acid, citramalic acid, fumaric acid, pyruvic acid) or were significantly lower in aerial roots (citric acid, homocitric acid, ketoglutaric acid, maleic acid, and malic acid). Some of these metabolites are part of the citric acid cycle and it can be speculated that the citric acid cycle is less prevalent in the aerial roots because it occurs mainly in the leaves. Isopropylmalic acid and suberic acid showed a deviating behavior, as their levels were higher in aerial roots. Although no significant effects were detected, hydroxy‐3‐methylbutyric acid showed the same trend.

#### 3.3.8 Amino acids and vitamins

Amino acids were present in all three plant organs with the exception of histidine which was not detected in aerial roots (Table 1). About one‐third of the amino acids showed no significant differences between the plant organs (e.g. proline, isoleucine, valine). Leucine, threonine, serine, and pipecolic acid were increased in aerial roots. Elevated leucine levels are supported by the higher amounts of its intermediates of biosynthesis (isopropylmalic acid and 3‐hydroxy‐3‐methylbutyric acid) (see section 3.3.7). Other metabolites including glutamine, glutamic acid, aspartic acid, and the aromatic amino acids (phenylalanine, tyrosine, tryptophan, histidine) generally showed higher intensities in internodes and/or leaves as compared to aerial roots. Individual amino acids exhibit various roles in plant physiology.^62^ Therefore, the observed differences between the plant organs may be interpreted in different ways and such derived hypotheses have to be proven in more specific studies and experiments.

Nicotinic acid was only detected in aerial roots, whereas tocopherols and pantothenic acid were detected in all plant organs. While for δ‐tocopherol no significant differences were observed, levels of α‐tocopherol were significantly increased in leaf samples. The higher phytol levels in leaves (see section 3.3.3) fits well with this, as phytyl pyrosphosphate is an intermediate of tocopherol biosynthesis.^63^ Pantothenic acid was elevated in leaves and aerial roots as compared to internodes.

### 3.4 Concluding remarks

We investigated the metabolome of different organs of *V. planifolia* using two complementary metabolomic platforms (GC × GC–MS, LC‐QToF‐MS). To the best of our knowledge, this is the first study to comprehensively characterize the metabolomes of different organs of the vanilla plant, i.e. leaves, internodes, and aerial roots (for images see Supporting Information Figure S8). Based on chromatographic and mass spectral data from in‐house databases or curated external spectral libraries, a total of 127 metabolites were identified (96 by means of level 1* confidence). Compared with an NMR metabolomic study, in which 36 distinct metabolites were reported,^13^ in this study the metabolite coverage was significantly enhanced (Figure 2). The characterized metabolite pattern comprises a large number of sugars, amino acids, fatty acids, and amines, but also vanilla‐specific secondary plant metabolites such as flavonoids (flavones), vanillin‐related metabolites, or terpenoids. Using PCA (Figure 1) and univariate statistics clear differences between the plant organs were observed. With respect to univariate statistical analysis 98 (of 127) metabolites (77%) showed significant differences, of which 16 metabolites were unique, i.e. not detected in at least one plant organ (Table 1; Supporting Information Table S2). Among other effects, it was striking that aglycons of flavonoids (Figure 4) and vanillin‐related metabolites (Figure 3) exhibited higher intensities in aerial roots, whereas its *O*‐glycoside forms tended to be higher in leaves and/or internodes. This suggests that more bioactive forms (aglycones) may accumulate where they are preferably needed, e.g. to protect the plant against pathogens.^64^ In contrast, *O*‐glycosides probably serve as a storage pool in the leaves.^50^

The focus of our study was on the molecular characterization of the vanilla metabolome; in addition, the first approaches to the interpretation of the metabolite data were given. This might help to understand further physiological processes in vanilla and support further research towards optimization of vanilla plant cultivation. Future studies should address a comparison of the metabolite profile between vanilla pods and vanilla plant organs investigated in this study, e.g. to further elucidate the biosynthetic pathway of vanillin.

## CONFLICT OF INTEREST STATEMENT

The authors declare no competing financial interest.

## Supporting information


**Table S1:** Concentrations of internal standards used in the study for quality control.

**Table S2:** Metadata of identified metabolites in *V. planifolia* and observed differences between the different plant organs (leaves, aerial roots, and internodes).

**Data S3.** Detailed Information to Untargeted UHPLC‐QToF‐MS analysis.

Tables S4 and S5. Detailed Information to GCxGC‐MS analysis.

**Data S6.** Detailed information to metabolite identification procedure.

**Table S7:** Metabolites involved in vanillin biosynthetic pathway in *V. planifolia* .

**Figure S8:** Images of investigated vanilla plant material.

## Data Availability

The data sets analyzed in the current study are available on request from the corresponding author. The data supporting the results of this study are available in the Supplementary material to this article.
