# Supplementary material for: Comprehensive metabolome characterization of leaves, internodes, and aerial roots of Vanilla planifolia by untargeted LC–MS and GC × GC–MS
Source: Phytochem Anal. 2024 Jul 21;36(1):30–51. doi: 10.1002/pca.3414 (PMC11743222; doi:10.1002/pca.3414)
Supplement: Supplementary file 3 — Data S3. Detailed Information to Untargeted UHPLC‐QToF‐MS analysis. [file PCA-36-30-s007.pdf]

Supporting Information S3: Detailed Information to Untargeted UHPLC-QToF-MS analysis

Comprehensive metabolome characterization of leaves, internodes and aerial roots of Vanilla planifolia by untargeted LC-MS and GC×GC-MS

Falco Beer, Christoph H. Weinert, Johannes Wellmann, Silke Hillebrand, Jakob Peter Ley, Sebastian T. Soukup, Sabine E. Kulling

Additional information to section “2.4.1 Internal standard (IS) mix preparation”

Internal standards (IS) used:
D-[UL-$^{13}C_6$]mannitol, [UL-$^{13}C_6$-Fru]sucrose, [$^{13}C_5$, ^{15}N]glutamine, [$^{13}C_5$, ^{15}N]valine, [$^{13}C_9$, ^{15}N]phenylalanine, histamine-d_4, methyl-α-D-glucopyranoside, D-pinitol, methyl-deoxyribose, phenyl-β-D-glucopyranoside (IS mix 1) and 2-amino-6-chloropurine, trans-3,4-methylendioxycinnamic acid, 5-bromo-2-hydroxyhippuric acid, benzoic acid-d_5, ferulic acid-d_3, [$^{13}C_6$]trans-resveratrol, hexylamine, 2-(4-chlorphenyl)ethylamine (IS mix 2).
Stock solutions of single compounds (2.5–100 mM) were prepared either in aqueous solution (0.5 mM HCl in H_2O or pure H_2O; IS mix 1 compounds) or in organic solvent (methanol, ethanol or dimethyl sulfoxide; IS mix 2 compounds) depending on its solubility behavior. For preparing IS mix 1 (aqueous) and IS mix 2 (organic) corresponding volumes of the stock solutions were diluted with either water or methanol resulting in final analyte concentrations of 100–250 µM. These working solutions were freshly prepared for each measurements series (polarity) and stored at -20°C until further processing. Detailed information with regard to the authentic standards are summarized in the Supporting Information Table S1. In addition, a total IS mix solution in pure solvent without plant matrix serving as a control for device performance was freshly prepared for each measurement series. 4 µl of the working solution of IS mix 1 and of IS mix 2 were diluted by addition of 92 µl of methanol reaching final concentrations of 4–10 µM.

Additional information to section “2.4.3 UHPLC-QToF-MS measurements”

In order to achieve robust data acquisition, the sample sequence was structured as follows:
(i) At the beginning of each measurement series, five solvent blanks were injected in order to allow the elimination of background noise features in the later data processing step (see section 2.4.4).
(ii) Subsequently, 16 equilibration QC samples (EQC) were injected for equilibrating the measurement system (intensities).
(iii) Thereafter, study samples were measured in consecutive blocks of eight samples each comprising of one post calibration QC sample (directly after the infusion of the mass calibration solution; see above), two QC samples (for quality assurance and correction of drift effects; see section 2.4.5) and five study samples. Study samples were measured randomized.
(iv) Internal standard mix in solvent (see Supporting Information S3) was injected at the beginning and at the end of each measurement series for monitoring device performance before and after the measurement.
