# Supplementary material for: Comprehensive metabolome characterization of leaves, internodes, and aerial roots of Vanilla planifolia by untargeted LC–MS and GC × GC–MS
Source: Phytochem Anal. 2024 Jul 21;36(1):30–51. doi: 10.1002/pca.3414 (PMC11743222; doi:10.1002/pca.3414)
Supplement: Supplementary file 4 — Tables S4 and S5. Detailed Information to GCxGC‐MS analysis. [file PCA-36-30-s005.pdf]

Supporting Information Tables S4 and S5

Comprehensive metabolome characterization of leaves, internodes and aerial roots of *Vanilla planifolia* by untargeted LC-MS and GC×GC-MS

Falco Beer, Christoph H. Weinert, Johannes Wellmann, Silke Hillebrand, Jakob Peter Ley, Sebastian T. Soukup, Sabine E. Kulling

Supporting Information Table S4. GC×GC-MS analysis: Detailed extraction, evaporation and derivatization protocol

Extraction	
**Work step**	**Action**
1	Thaw study samples and QC sample aliquots on ice
2	Add 750 µL (leaf and internode samples) or 375 µL (aerial root samples) of methanol
3	Mix samples vigorously
4	Incubate samples for 10 min at 35 °C and 1,400 rpm in a shaker
5	Remove insoluble matter by short centrifugation in a small table centrifuge
6	Transfer 700 µL of the supernatants into new 2 mL reaction tubes
7	Re-extract the samples (repeat steps 2, 4 and 5)
8	Combine 700 µL of the second supernatant with the first one
9	Sediment insoluble matter by centrifugation for 3 min at 4 °C and 16100 × g
10	Transfer 30 µL of the combined supernatants to screw-capped glass vials with 300 µL micro inserts
11	Add 12 µL of a mixture of internal standards containing hexylamine (100 µM), 1-O-methyl-2-desoxy-D-ribose (100 µM), 2-chlorophenylacetic acid (100 µM), p-chloro-L-phenylalanine (40 µM), 3-O-methyl-D –glucopyranose (40 µM), 5-bromo-2,4-dihydroxybenzoic acid (40 µM) and phenyl-β-D-glucopyranoside (25 µM) in methanol/water to each vial
Evaporation	
**Work step**	**Action**
1	Dry samples in a rotary vacuum concentrator (RVC 2-25 CDplus/ Alpha 2-4 LSCbasic; Christ, Osterode, Germany) for 50 min at 40 °C and a final vacuum of <0,1 mbar
2	Re-dissolve samples again in 20 µL of methanol
3	Dry samples again to remove traces of water (30 min, 40 °C, <1 mbar)
Derivatization	
**Work step**	**Action**
1	Add 20 µL of methoxylamine-hydrochloride in pyridine (20 mg/mL)
2	Incubate for 1 h at 40 °C and 1,000 rpm in a shaker
3	Add 50 µL of MSTFA with 1 % TMCS
4	Incubate for 1 h at 65 °C and 1,000 rpm in a shaker
5	Add 10 µL of each retention index marker mixtures (alkanes, FAMEs) only to the daily RI-QC sample

Supporting Information Table S5. GC×GC-MS analysis: Instrumental setup and settings

GC×GC method	
Carrier gas	Helium 5.0
GC mode	Constant velocity
Purge flow	6 mL/min
Initial column head pressure	249.9 kPa
Liner type	Deactivated split liner with quartz wool
Primary column	Rxi-5SilMS, ^1L = 20 m plus 5 m of a pre-column, ^1d_c = 0.18 mm, ^1d_f = 0.18 µm (Restek, Bellefont, USA)
Secondary column	BPX50, $^2L_{total}$ = 2.2 m, including a “separation segment” of $^2L_{sep}$ = 0.7 m, ^2d_c = 0.15 mm, ^2d_f = 0.15 µm (Trajan Scientific, Ringwood Victoria, Australia)
Column connector	µ-Union (Trajan Scientific, Ringwood Victoria, Australia)
GC temperature ramp	90 °C → 3.5 °C/min → 200 °C → 6 °C/min → 310°C → 35 °C/min → 345 °C (hold 3.74 min)
Run time	54.5 min
Injection mode	Hot split
Injector temperature	250 °C
Split ratio program	1:3 (hold 1 min) → 1:20 (hold 4 min) → 1:3 (hold until end of run)
Injection volume	1.0 µL
Injector syringe	10 µL, with PTFE-tipped plunger (Trajan Scientific, Ringwood Victoria, Australia)
Syringe wash solvent 1	Acetone
Syringe wash solvent 2	Heptane
Modulator settings	
Modulation period (PM)	3.0 s
Cold jet temperature	-90 °C
Hot jet temperature program	200 °C (hold until 15 min) → 250 °C (hold until 30 min) → 300 °C (hold until 40 min) → 380 °C (hold until 53 min) → 200 °C (hold until end of run)
Hot jet pulse duration	350 ms

Supporting Information Table S5. GC×GC-MS analysis: Instrumental setup and settings (continued).

MS settings	
Interface temperature	290 °C
Ion source temperature	250 °C
Ionization mode	EI (70 eV)
MS Mode	Scan
Scan speed	20.000 u/s
Scan range	m/z 60-374
Event time	20 ms
Data acquisition frequency	50 s^{-1}
Data acquisition period	2.7 -54.5 min
Detector voltage windows	02.70 – 12.56 min: 1.01 kV<br>12.56 – 13.41 min: 0,92 kV<br>13.41 – 24.06 min: 1.01 kV<br>24.06 – 26.01 min: 0.92 kV<br>26.01 – 39.76 min: 1.01 kV<br>39.76 – 45.31 min: 0.92 kV<br>45.31 – 54.50 min: 1.01 kV
