# Supplementary material for: Comprehensive metabolome characterization of leaves, internodes, and aerial roots of Vanilla planifolia by untargeted LC–MS and GC × GC–MS
Source: Phytochem Anal. 2024 Jul 21;36(1):30–51. doi: 10.1002/pca.3414 (PMC11743222; doi:10.1002/pca.3414)
Supplement: Supplementary file 5 — Data S6. Detailed information to metabolite identification procedure. [file PCA-36-30-s006.pdf]

Supporting Information S6: Detailed information to metabolite identification procedure

Comprehensive metabolome characterization of leaves, internodes and aerial roots of Vanilla planifolia by untargeted LC-MS and GC×GC-MS

Falco Beer, Christoph H. Weinert, Johannes Wellmann, Silke Hillebrand, Jakob Peter Ley, Sebastian T. Soukup, Sabine E. Kulling

Additional Information to section "2.6.1 Metabolite identification", subsection "Metabolite identification based on LC-MS metabolomics"

Reference compounds were measured under the same UHPLC-QToF-MS conditions as the samples of this study. For all features annotated by MS-DIAL via the in-house database the chromatographic peaks and corresponding MS data were checked. At the first step in identification process only features annotated by MS-DIAL post-identification remained in the feature list which fulfil the following criteria: total score > 80, RT similarity > 80, ΔRT (sample vs. reference) ≤ ±0.1 min, Δm/z precursor ion (sample vs. reference) ≤ ±10 mDa and signal-to-noise ratio (SN) > 10. The total score was calculated by the MS-DIAL software based on retention time and m/z similarity considering the acquired exact mass or retention time and the respective reference value from the inhouse database as well as a retention time tolerance of 0.1 min and an MS1 mass tolerance of 0.015 Da, respectively. For details see MS-Dial instructions.
In a second step, an automated MS2 spectral matching of the acquired spectrum in the vanilla dataset was performed with the in-house spectral library using the ´Quantitation and Targeted Identification´ tool in Sciex OS (version 1.6). Matching results were evaluated visually and by including software-calculated scores, i.e. purity score (considering all signals from sample and reference spectrum), fit (only considering the signals from sample spectrum) and reverse fit (only considering the signals found in the reference spectrum).
Ten compounds, which are part of the biosynthesis pathway of vanillin and/or were identified in V. planifolia by NMR,[1-8] namely vanillic acid 4-*O*-glucoside, ferulic acid 4-*O*-glucoside, vanillin, glucovanillin, 3,4-dihydroxybenzaldehyde, 4-hydroxybenzaldehyde, p-hydroxybenzylalcohol, p-hydroxybenzylalcohol glucoside, 3,4-dimethoxycinnamic acid and homocitrate, were not part of the initially used in-house database file used for post-identification in MS-DIAL. Their corresponding retention time values as well as MS and MS2 data were obtained by post-measurements of the reference compounds. These compounds were identified in the vanilla data set by manually searching for the respective accurate masses and retention times in the raw feature list (MS-DIAL) and, subsequently, by spectral data matching via Sciex OS, as described above.
In order to facilitate metabolite identification post-measurements were performed with the aim to enhance the proportion of features with MS2 spectra of sufficient quality. Therefore, two QC samples were prepared, but in contrast to the standard protocol, the resulting filtrates were concentrated under nitrogen flow until reaching a final sample volume of approx. 100 µl (corresponding to a concentration factor of ca. 5). In addition, individual samples were re-processed and re-analyzed with the aim to obtain MS2 spectra with higher qualities for some selected analytes in order to enhance their identification.

References:

(1) Leyva VE, Lopez JM, Zevallos-Ventura A, Cabrera R, Cañari-Chumpitaz C, Toubiana D, Maruenda H. In vitro selection of vanilla plants resistant to *Fusarium oxysporum* f. sp. *vanillae*. *Food Chem* 2021, 358:129365-129374.
(2) Kundu A. Vanillin biosynthetic pathways in plants. *Planta* 2017, 245:1069-1078.
(3) Gallage NJ, Hansen EH, Kannangara R, Olsen CE, Motawia MS, Jørgensen K, Holme I, Hebelstrup K, Grisoni M, Møller BL. Vanillin–bioconversion and bioengineering of the most popular plant flavor and its de novo biosynthesis in the Vanilla orchid. *Nat Commun* 2014, 5:4037-4050.
(4) Brodelius PE. *Phytochem Anal* 1994, 5:27-31.
(5) Funk C, Brodelius PE. Phenylpropanoid metabolism in suspension cultures of *Vanilla planifolia* Andr. *Plant Physiol* 1990, 94:95-101.
(6) Funk C, Brodelius, PE. Phenylpropanoid metabolism in suspension cultures of *Vanilla planifolia* Andr. *Plant Physiol* 1990, 94:102-108.
(7) Pak FE, Gropper S, Dai WD, Havkin-Frenkel D, Belanger FC. Characterization of a multifunctional methyltransferase from the orchid *Vanilla planifolia*. *Plant Cell Rep* 2004, 22:959-966.
(8) Podstolski A, Havkin-Frenkel D, Malinowski J, Blounta JW, Kourteva G, Dixon RA. A re-evaluation of the final step of vanillin biosynthesis in the orchid *Vanilla planifolia*. *Phytochemistry* 2002, 61:611-620.
