# Supplementary material for: Comprehensive metabolome characterization of leaves, internodes, and aerial roots of Vanilla planifolia by untargeted LC–MS and GC × GC–MS
Source: Phytochem Anal. 2024 Jul 21;36(1):30–51. doi: 10.1002/pca.3414 (PMC11743222; doi:10.1002/pca.3414)
Supplement: Supplementary file 7 — Figure S8: Images of investigated vanilla plant material. [file PCA-36-30-s001.pdf]

Supporting Information Figure S8: Images of investigated vanilla plant material

Comprehensive metabolome characterization of leaves, internodes and aerial roots of *Vanilla planifolia* by untargeted LC-MS and GC×GC-MS

Falco Beer, Christoph H. Weinert, Johannes Wellmann, Silke Hillebrand, Jakob Peter Ley, Sebastian T. Soukup, Sabine E. Kulling

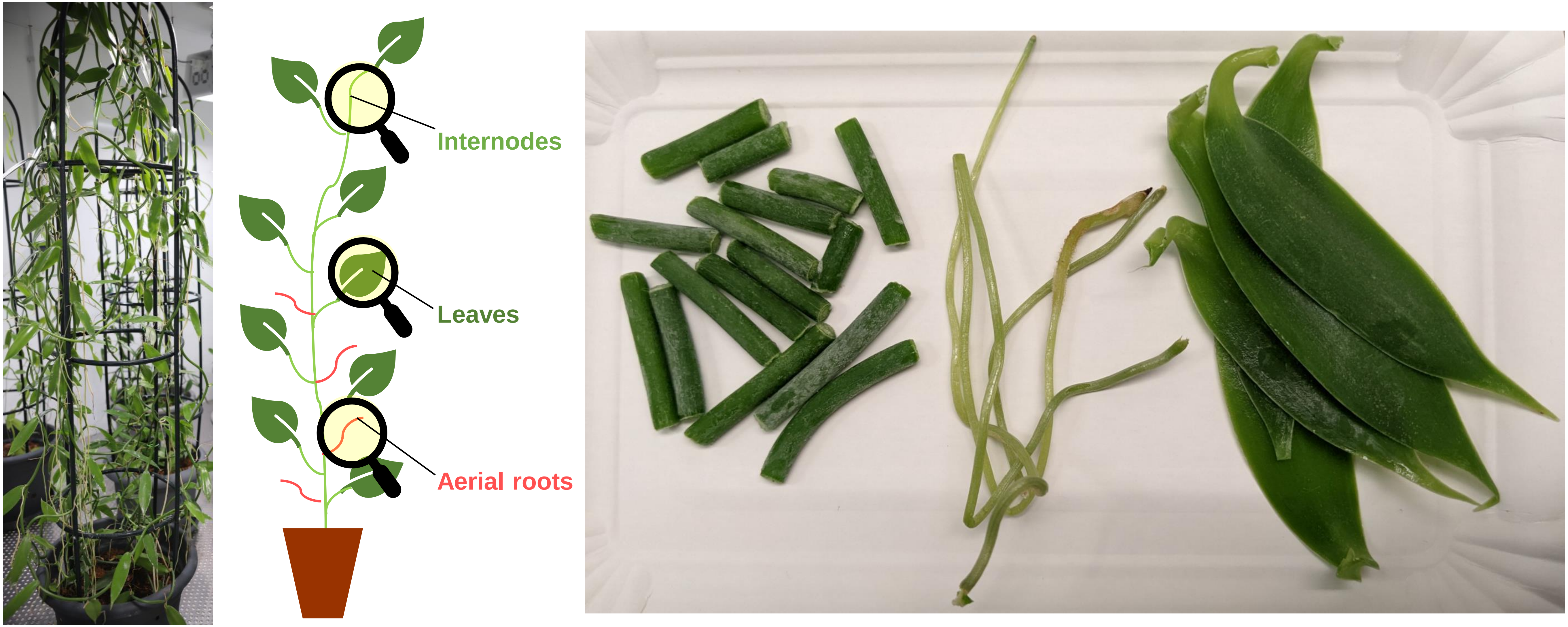

Vanilla planifolia Internodes Aerial roots Leaves
